# TGFBI Maintains Fibrocartilage Homeostasis by Restraining O‐GlcNAcylation to Inactivate and Destabilize ADAMTS16 in Temporomandibular Joint Osteoarthritis

**DOI:** 10.1002/advs.77594

**Published:** 2026-09-16

**Authors:** Xin Liu, Xinyue Luo, Jie Zhao, Ziyan Jiang, Jiayi Zhai, Taomin Zhu, Xueke Jia, Xiaohan Ma, Henghua Jiang, Huimin Li, Yaping Feng, Jin Ke, Xing Long

**Affiliations:** ^1^ State Key Laboratory of Oral & Maxillofacial Reconstruction and Regeneration Hubei Key Laboratory of Stomatology Key Laboratory of Oral Biomedicine Ministry of Education School & Hospital of Stomatology Wuhan University Wuhan China; ^2^ Department of Oral and Maxillofacial Surgery School & Hospital of Stomatology Wuhan University Wuhan China

**Keywords:** adamts, cell biology, condyle, extracellular matrix, ex vivo, homeostasis, perichondrium, protein degradation, protein turnover, senescence

## Abstract

The mandibular condyle is covered by fibrocartilage, and its degeneration, termed temporomandibular joint osteoarthritis (TMJOA), currently lacks effective disease‐modifying therapies. In this study, we uncover a previously unrecognized mechanism by which the extracellular matrix protein TGFBI (TGF‐β‐induced) sustains condylar fibrocartilage homeostasis by restraining protein O‐GlcNAcylation. We demonstrate that TGFBI is predominantly expressed in the condylar perichondrium and is markedly downregulated in both TMJOA patients and corresponding mouse models. Genetic ablation of *Tgfbi* delays postnatal skeletal development, promotes chondrocyte hypertrophy, and accelerates fibrocartilage degeneration. Mechanistically, TGFBI functions as a negative regulator of O‐GlcNAcylation in condylar perichondrial cells (cPCs), thereby suppressing both the transcriptional activation and protein stability of the matrix protease ADAMTS16. Notably, ex vivo studies identify Ser1170 of ADAMTS16 as a critical O‐GlcNAcylation site that directly governs its protein turnover. By inhibiting O‐GlcNAcylation at this residue, TGFBI prevents condylar chondrocyte hypertrophy, senescence, and extracellular matrix degradation. Collectively, our findings establish the TGFBI–O‐GlcNAcylation–ADAMTS16 axis as a vital driver of condylar fibrocartilage degeneration and highlight a promising metabolic target for TMJOA therapy.

## Graphical abstract

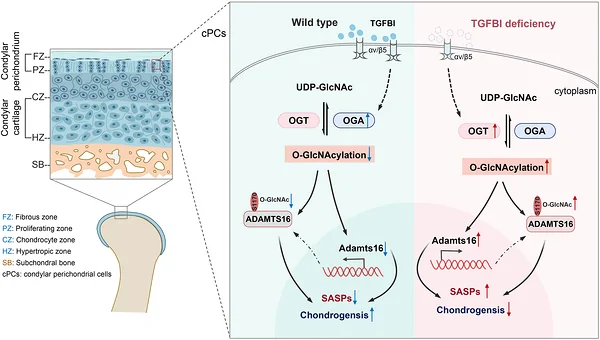

## 1 Introduction

Temporomandibular joint osteoarthritis (TMJOA) is considered the most severe form of temporomandibular joint disorder (TMD). It is characterized by the progressive degradation of articular cartilage, subchondral bone sclerosis, and inflammation of the synovial membrane [1, 2, 3]. TMJOA leads to chronic pain, which profoundly compromises patients' quality of life [4, 5, 6]. The articular surface of the mandibular condyle is covered by fibrocartilage. This specialized tissue, which includes knee meniscus, intervertebral disc, and temporomandibular joint (TMJ) disc, is characterized by a composite extracellular matrix (ECM) that consists of both fibrous and cartilaginous components [7, 8, 9, 10, 11]. The balance of these components varies based on the specific biomechanical demands of different tissues. Collagen‐rich fibrous tissue provides tensile strength, while proteoglycan‐enriched cartilaginous tissue offers compressive resistance.

In the postnatal mandible, the condylar cartilage is organized into four distinct histological zones [12, 13, 14]. The most superficial layer consists of a perichondrium‐like tissue that lines the articular surface and contains a population of condylar perichondrial cells (cPCs). This layer can be further subdivided into two distinct regions: an outer fibrous zone (FZ) and an inner proliferating zone (PZ) [15, 16]. Beneath the perichondrium lies the condylar cartilage proper, which consists of a chondrocyte zone (CZ) with flattened chondrocytes and a deeper hypertrophic zone (HZ) filled with hypertrophic chondrocytes. Due to its crucial role as a reservoir for progenitor cells and its function in cartilage maintenance and repair, the condylar perichondrium serves as a promising therapeutic target for treating trauma and degenerative diseases affecting fibrocartilage [17, 18, 19].

The extracellular matrix protein transforming growth factor‐β‐induced (TGFBI) is strongly associated with both amyloid and amorphous protein deposition in corneal dystrophies [20, 21, 22]. Although TGFBI is a recognized transcriptional target of TGF‐β, the specific functional roles of TGFBI in chondrocytes and cartilage homeostasis remain inadequately defined [23, 24, 25]. Under physiological conditions, TGFBI may enhance beneficial communication between cells and the ECM. This interaction has the potential to promote chondrocyte adhesion and survival, support ECM synthesis and homeostasis, and contribute to the stability of cartilage tissue [26, 27, 28]. However, in the degenerative microenvironment of osteoarthritis (OA), TGFBI may contribute to disease progression by disrupting normal matrix synthesis and promoting catabolic responses [29, 30, 31]. Given the structural specificity of the temporomandibular joint, where the unique biological and biomechanical properties of condylar fibrocartilage may lead to distinct performances of TGFBI. Therefore, it is essential to elucidate the precise mechanisms of TGFBI in the pathogenesis of TMJOA to assess its potential as a therapeutic target.

Cellular responses to ECM‐derived cues and various stress signals are intricately regulated by post‐translational modifications, including O‐linked β‐N‐acetylglucosaminylation (O‐GlcNAcylation) [32, 33]. This unique and dynamic monosaccharide modification serves as both a nutrient and stress sensor by integrating metabolic flux through the hexosamine biosynthesis pathway [34]. This modification is catalyzed by O‐GlcNAc transferase (OGT) and removed by O‐GlcNAcase (OGA) [35]. O‐GlcNAcylation serves as a central metabolic rheostat, directly influencing transcriptional activity, stress responses, and protein function in articular chondrocytes [36, 37, 38]. Perturbations in O‐GlcNAcylation homeostasis are increasingly linked to the pathogenesis of OA. Elevated levels of O‐GlcNAcylation have been shown to accelerate chondrocyte apoptosis, ferroptosis, and inflammatory responses [39, 40, 41, 42]. We hypothesize that TGFBI may modulate key protein post‐translational modifications, such as O‐GlcNAcylation, either directly or indirectly. This modulation could integrate cellular metabolic status with ECM composition and overall cartilage health—a connection that has not yet been explored.

In this study, we reveal a novel function for TGFBI in the mandibular condylar perichondrium. We demonstrate that its predominant expression in this area modulates the homeostasis of cPCs by suppressing global protein O‐GlcNAcylation. This suppression further regulates chondrocyte hypertrophy, senescence, and cartilage degeneration. This newly identified mechanistic axis not only enhances our understanding of TMJOA pathobiology but also provides a promising foundation for developing targeted, disease‐modifying therapies aimed at biologically restoring the mandibular condylar fibrocartilage.

## 2 Results

### 2.1 Down‐Regulation of TGFBI in Both TMJOA Patients and Experimental Mouse Models

**FIGURE 1:**
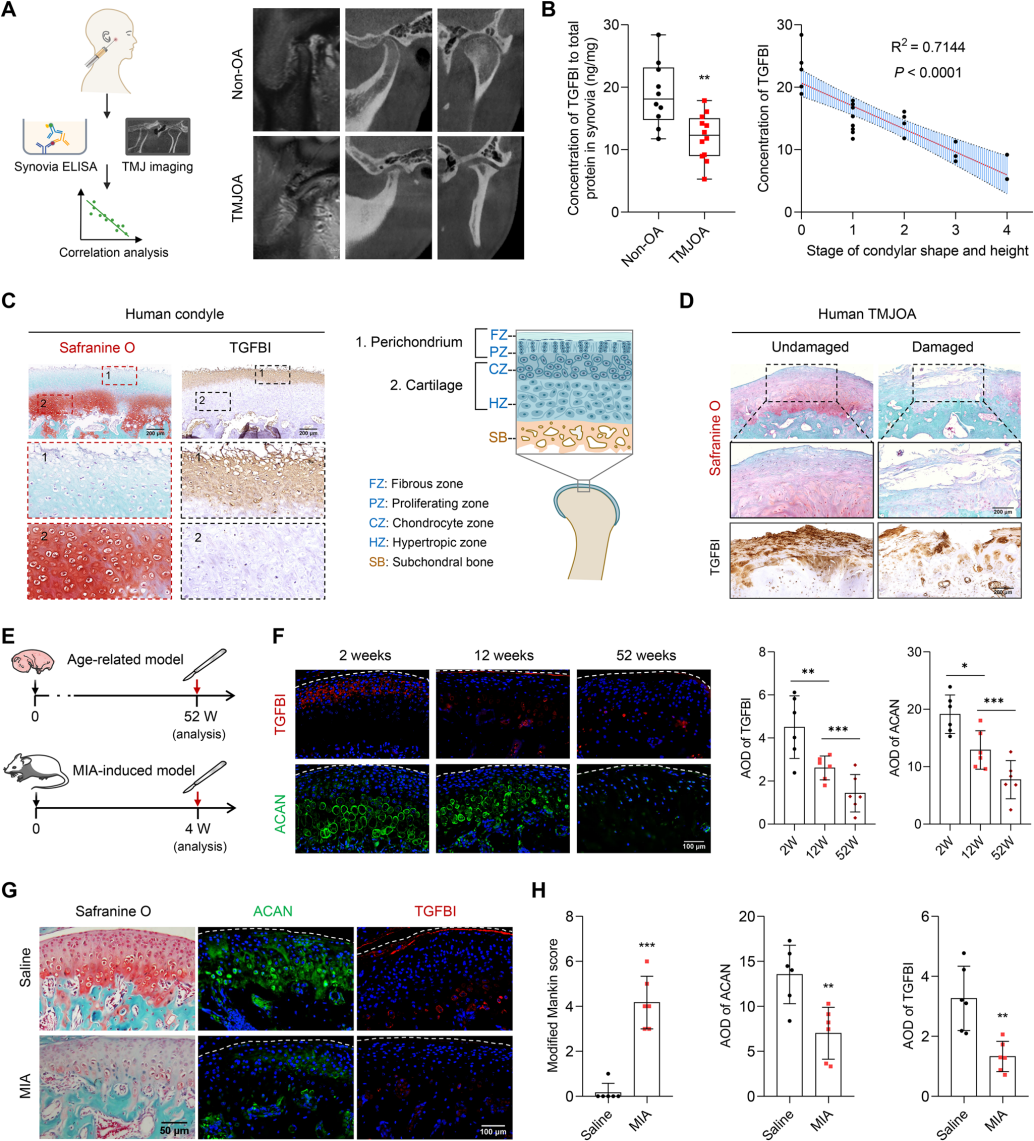
Downregulation of TGFBI in TMJOA tissues from patients and mice. (A) Flowchart illustrating the analysis of synovial TGFBI levels and TMJ imaging in TMD patients. Representative MRI and CBCT images are shown. (B) Left panel: ELISA quantification of TGFBI concentration (normalized to total protein) in synovial fluid from TMD patients, unpaired t‐test, n = 10 for Non‐OA, n = 12 for TMJOA. Right panel: Correlation analysis between synovial fluid TGFBI levels and radiographic staging scores of condylar degenerations in TMD patients, linear regression analysis, data are presented as linear regression fit and 95% confidence interval, n = 22. (C) Safranine O staining and immunohistochemical staining of TGFBI in human condylar cartilage from condylar fracture patients. Schematic diagram on right depicts the layered structure of the condyle: fibrous zone (FZ), proliferating zone (PZ), chondrocyte zone (CZ), hypertrophic zone (HZ), and subchondral bone (SB). (D) Safranine O staining and TGFBI immunostaining comparing undamaged and damaged regions of condylar cartilage from TMJOA patients. (E) Experimental schematics for the age‐related and MIA‐induced TMJOA mouse models. (F) Immunofluorescence staining and quantification of TGFBI and ACAN expression in condylar cartilage from age‐related mice, one‐way ANOVA, n = 6. (G) Safranine O staining and immunohistochemical staining for ACAN and TGFBI in condylar cartilage from MIA‐induced TMJOA mice. (H) Quantification of modified Mankin score, and average optical density (AOD) of ACAN and TGFBI staining in (G), unpaired t‐test, n = 6. Unless otherwise specified, data are presented as mean ± SD; *p < 0.05, **p < 0.01, ***p < 0.001.

To establish the clinical relevance of TGFBI in TMJOA, we began our investigation by profiling its expression in human disease samples (Figure 1A) and complementary mouse models (Figure 1E). Our ELISA analysis of synovial fluid from TMD patients revealed a significant reduction in TGFBI protein concentration in the TMJOA group compared to the non‐OA control group (Figure 1B). Furthermore, the level of TGFBI in the synovial fluid of TMD patients showed a clear inverse correlation with the radiological staging score of condylar degeneration (Figure 1B), indicating that lower TGFBI levels are associated with more advanced structural damage in the TMJ. Histological examination of human condylar cartilage specimens localized TGFBI protein primarily within the superficial fibrous zone (FZ) and the adjacent proliferating zone (PZ), which are key components of the mandibular condylar perichondrium (Figure 1C). Crucially, in condylar cartilage samples from TMJOA patients, areas showing visible degradation and matrix loss exhibited markedly reduced TGFBI immunostaining intensity compared to preserved, undamaged regions from the same patients (Figure 1D). Integrin αV/β5, one of TGFBI's membrane‑bound receptors, has been confirmed as the key integrin receptor in fibrocartilage [43]. In the present study, integrin αV/β5, but not integrin αV/β3, exhibited major expression in undamaged human condylar fibrocartilage, and were decreased in damaged areas (Figure S1A). These manifestations collectively suggest that lower concentration of TGFBI may lead to more severe degeneration of the condylar fibrocartilage in the TMJ.

This pattern was also faithfully replicated in our animal models of mice. In age‐related degenerative mice, a natural model of joint degeneration [44, 45], we observed significant time‐dependent co‐downregulation of TGFBI and the essential cartilage matrix component aggrecan (ACAN) in the condylar cartilage (Figure 1F). Similarly, in the MIA‐induced chemical model of TMJOA, the onset of mouse cartilage degeneration was confirmed by elevated modified Mankin OA scores and reduced Safranine O staining within condylar cartilage, along with a pronounced decrease of TGFBI and ACAN protein expression within condylar cartilage (Figure 1G,H). Single‐cell RNA sequencing analysis further revealed that *Itgb5* and *Itgav* were predominantly expressed in mouse chondrocyte subpopulations, whereas *Itgb3* expression was barely detectable in these cells (Figure S1B–D). Collectively, these findings across species and models strongly indicate that diminished TGFBI expression within the condylar fibrocartilage is a key feature associated with the pathogenesis of TMJOA.

### 2.2 *Tgfbi* Deficiency Delays Postnatal Skeletal Development and Exacerbates Age‐Related TMJ Degeneration in Mice

**FIGURE 2:**
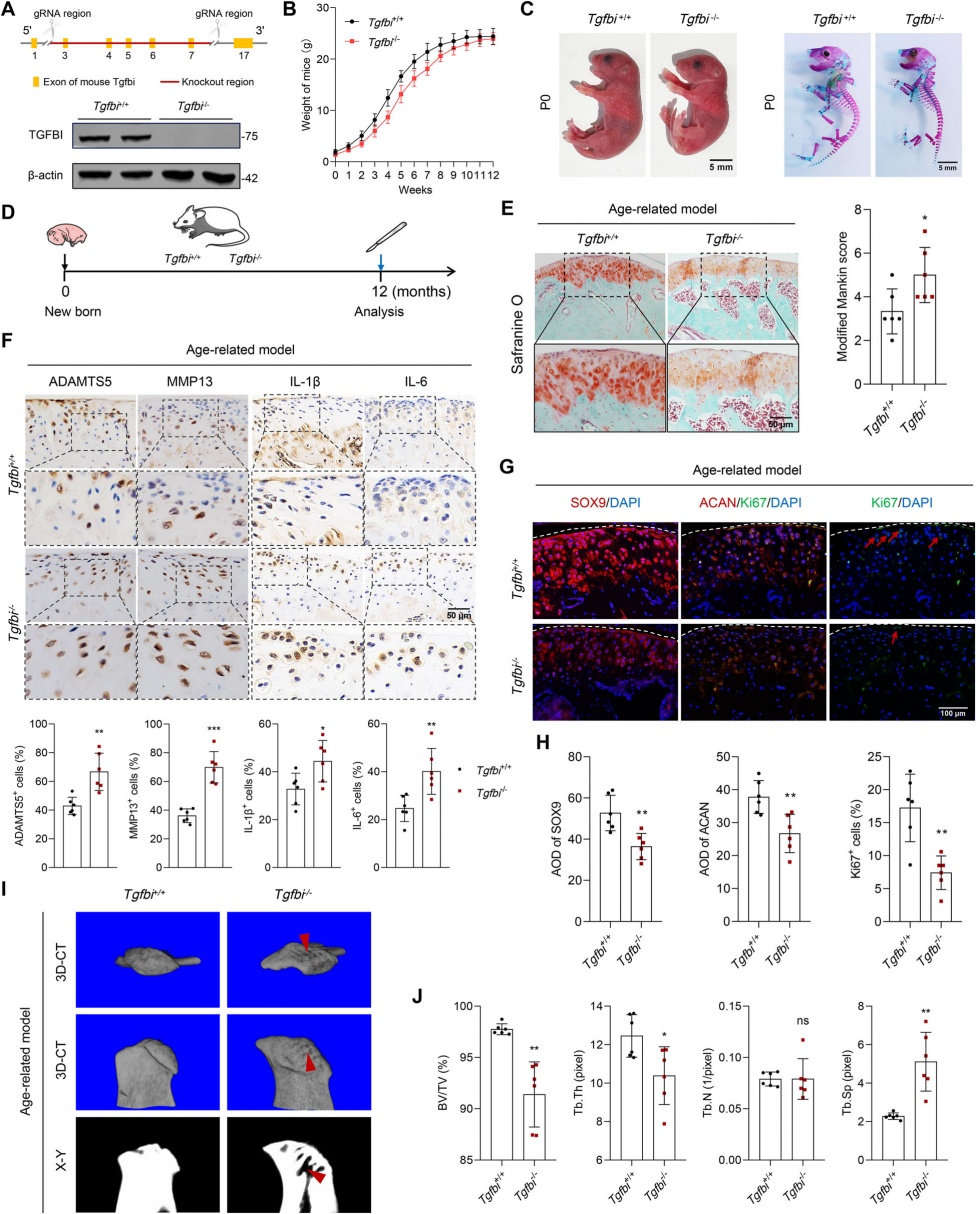
*Tgfbi* deficiency impairs skeletal development and exacerbates age‐related TMJOA in mice. (A) Schematic of the Tgfbi knockout strategy, and confirmation of TGFBI protein depletion in *Tgfbi* ^−/−^ mice. (B) Body weight curves of *Tgfbi* ^+/+^ and *Tgfbi* ^−/−^ mice during postnatal growth, n = 6. (C) Gross morphology and alcian blue/alizarin red skeletal staining of newborn mice. (D) Flowchart for the analysis of TMJ tissues from 12 months age‐related *Tgfbi* ^+/+^ and *Tgfbi* ^−/−^ mice. (E) Safranine O staining and quantification of modified Mankin score within mouse condylar cartilage, unpaired t‐test, n = 6. (F) Immunohistochemical staining and quantification of catabolic mediators (ADAMTS5, MMP13) and senescence‐associated secretory phenotypes (IL‐1β, IL‐6) in mouse condylar cartilage, unpaired t‐test, n = 6. (G) Immunofluorescence staining of SOX9, and co‐staining of ACAN with Ki67 in mouse condylar cartilage. (H) Quantification analyzing AOD of SOX9 and ACAN in mouse condylar cartilage, and percentage of Ki67‐positive cells within mouse condylar perichondrium, unpaired t‐test, n = 6. (I, J) Representative micro‐CT images (I) and quantitative analysis (J) of condylar subchondral bone microstructure, unpaired t‐test, n = 6. Data are presented as mean ± SD; *p < 0.05, **p < 0.01, ***p < 0.001; ns, not significant.

To directly investigate the functional role of TGFBI in vivo, we utilized a constitutive *Tgfbi* knockout (KO) mouse model (Figure 2A). Successful genomic editing and the absence of TGFBI protein were confirmed through Sanger sequencing, genotyping PCR, and immunoblot (Figure S2A–C, Figure 2A). Phenotypic analysis showed that *Tgfbi* ^−/−^ neonates exhibited a dwarfism and consistently lagged in body weight gain during the early postnatal period compared to their wild‐type (WT) littermates (Figure 2B,C). Specifically, whole‐mount skeletal staining of newborns with alcian blue (for cartilage) and alizarin red (for bone) revealed noticeable delays in ossification and cartilage maturation in KO mice, indicating an important role for TGFBI in skeletal morphogenesis (Figure 2C).

We then assessed the long‐term effects of *Tgfbi* deficiency on joint integrity by examining the age‐related joint degenerative phenotype in 12‐month‐old mice (Figure 2D). Histopathological evaluation using Safranine O/Fast Green staining demonstrated significantly more severe condylar cartilage erosion and loss of proteoglycan staining in 12‐month‐old *Tgfbi* ^−/−^ mice than age‐matched WT controls, resulting in a higher modified Mankin score (Figure 2E). While overall condylar cartilage thickness was not drastically altered, there was a noted trend toward reduced chondrocyte cellularity in age‐related KO mice (Figure S2D, E). Immunohistochemistry revealed a significantly pro‐catabolic and inflammatory senescent microenvironment in the condylar cartilage of age‐related KO mice. This environment is marked by increased levels of the matrix‐degrading enzymes ADAMTS5 and MMP13, along with elevated production of the senescence‐associated secretory phenotype (SASP) factors IL‐1β and IL‐6 (Figure 2F). Concurrently, markers of cartilage anabolism and homeostasis, including the master transcription factor SOX9 and the extracellular matrix protein ACAN, were suppressed, alongside a decreased number of Ki67‐positive proliferating chondrocytes (Figure 2G,H). Consistently, single‐cell transcriptomic analysis revealed that the expression of *Itgb5* and *Itgav*, but not *Itgb3*, was enriched in chondrocyte clusters from WT mice, with a noticeable reduction in *T* *gfbi*‐deficient chondrocytes (Figure S2F). This degenerative condylar cartilage tissue pathology was accompanied by a significant deterioration of the underlying subchondral bone. Micro‐computed tomography (µCT) analysis revealed that age‐related *Tgfbi* KO mice exhibited an osteoporotic‐like changes in the mandibular condyle, with significant reductions in BV/TV and Tb. Th, and an increase in Tb. Sp (Figure 2I,J). These data clearly demonstrate that *Tgfbi* deficiency compromises both developmental skeletal growth and the maintenance of TMJ fibrocartilage homeostasis as aging occurs, resulting in accelerated OA‐like degeneration.

### 2.3 *Tgfbi* Ablation Promotes a Hypertrophic Shift in Condylar Chondrocyte Populations

**FIGURE 3:**
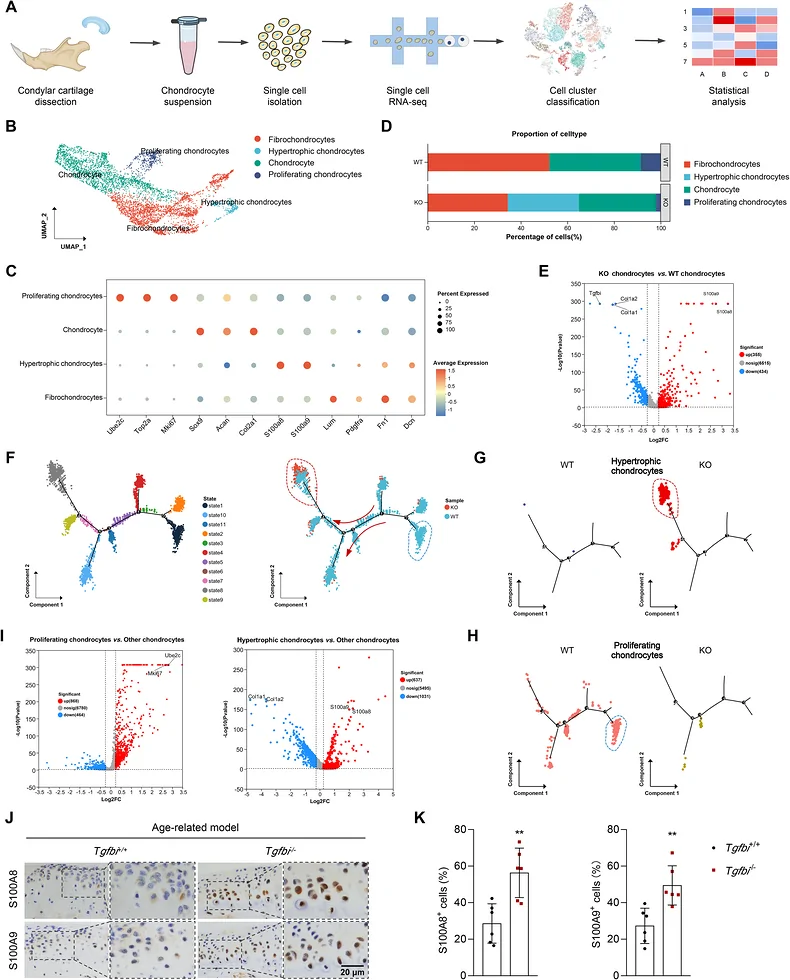
*Tgfbi* knockout promotes hypertrophic degeneration of condylar chondrocytes in mice. (A) Workflow for single‐cell isolation, scRNA‐seq, and bioinformatic analysis of condylar cartilage from 8‐week‐old WT and *Tgfbi* ^−/−^ mice. (B) UMAP visualization of four chondrocyte subpopulations: fibrochondrocytes, proliferating chondrocytes, chondrocyte, and hypertrophic chondrocytes. (C) Bubble plot displaying the feature gene expression for each chondrocyte subpopulation. (D) Stacked bar chart comparing the proportional distribution of four chondrocyte subpopulations between WT and KO groups. (E) Volcano plot highlighting the DEGs in condylar chondrocytes from WT versus KO mice. (F) Pseudotime trajectory analysis (Monocle2) of chondrocyte subtypes, colored by cell state or genotype. (G, H) Pseudotime trajectories for proliferating chondrocytes (G) and hypertrophic chondrocytes (H) in WT and KO mice. (I) Volcano plots showing gene expression differences in proliferating chondrocytes or hypertrophic chondrocytes compared to other subpopulations. (J, K) Immunohistochemical staining (J) and quantification (K) of degenerative markers S100A8 and S100A9 in condylar cartilage from age‐related WT and KO mice, unpaired t‐test, n = 6. Data are presented as mean ± SD; **p < 0.01. WT, wild type; KO, knockout; DEGs, differentially expressed genes.

To elucidate the cellular mechanisms underlying the TMJOA phenotype in *Tgfbi*‐deficient mice, we conducted single‐cell RNA sequencing (scRNA‐seq) on condylar cartilage and surrounding tissues obtained from 8‐week‐old WT and *Tgfbi* KO mice (Figure 3A). Initial clustering of all cells identified major populations, including chondrocytes, endothelial cells, immune cells, and others (Figure S3A). We subsequently focused our analysis on chondrocyte populations, which were identified by canonical markers *Col2a1*, *Sox9*, *Acan*, and *Comp* (Figure S3B). Eight clusters were identified within the chondrocyte populations, specifically clusters 3, 4, 10, 11, 14, 15, 18, and 19 (Figure S3C). Based on established and newly identified marker genes, we annotated the chondrocyte populations into four biologically relevant subpopulations: fibrochondrocytes of the superficial layer, proliferating chondrocytes, chondrocyte zone cells, and hypertrophic chondrocytes (Figure 3B,C). Comparative analysis revealed a significant alteration in chondrocyte composition following *Tgfbi* loss: the proportion of hypertrophic chondrocytes (cluster 18) was significantly increased, while the pool of proliferating chondrocytes (cluster 15) correspondingly decreased in KO mice compared to WT controls (Figure 3D, Figure S3C, D). Differential gene expression analysis highlighted the enrichment of genes associated with cartilage degradation, loss of fibrous characteristics, and hypertrophic differentiation in chondrocytes from KO mice (Figure 3E).

Pseudotime trajectory analysis using Monocle2 further supported these findings, indicating that *Tgfbi* deficiency altered the dynamics of chondrocyte differentiation. In KO mice, chondrocytes followed a skewed developmental path that favored progression toward a hypertrophic fate, while showing a significant reduction in the proliferating chondrocyte branch (Figure 3G,H, Figure S3G,H). Examination of subtype‐specific markers confirmed high expression of proliferation‐associated genes (*Mki67*, *Ube2c*, *Top2a*) in proliferating chondrocytes, alongside robust expression of the pro‐inflammatory and hypertrophy‐linked genes (*S100a8*, *S100a9*), as well as decreased expression of fibrous genes (*Col1a1*, *Col1a2*) in hypertrophic chondrocytes (Figure 3I, Figure S3E, F). Notably, the expression of *Itgb5* and *Itgav* was primarily localized in fibrochondrocytes and proliferating chondrocytes, whereas *Itgb3* showed minimal expression across all chondrocyte subtypes (Figure S3I). In line with the transcriptomic data, immunohistochemical staining of condylar cartilage from age‐related mice exhibited elevated protein levels of the hypertrophy markers S100A8 and S100A9, particularly in the hypertrophic zone of mouse condylar cartilage, in *Tgfbi* ^−/−^ mice compared to WT controls (Figure 3J,K). Thus, the loss of *Tgfbi* fundamentally alters the fate decisions of chondrocytes within the condyle, leading to a pathological shift toward hypertrophic degeneration of condylar chondrocytes.

### 2.4 rhTGFBI Promotes Chondrogenesis and Mitigates Senescence in cPCs

**FIGURE 4:**
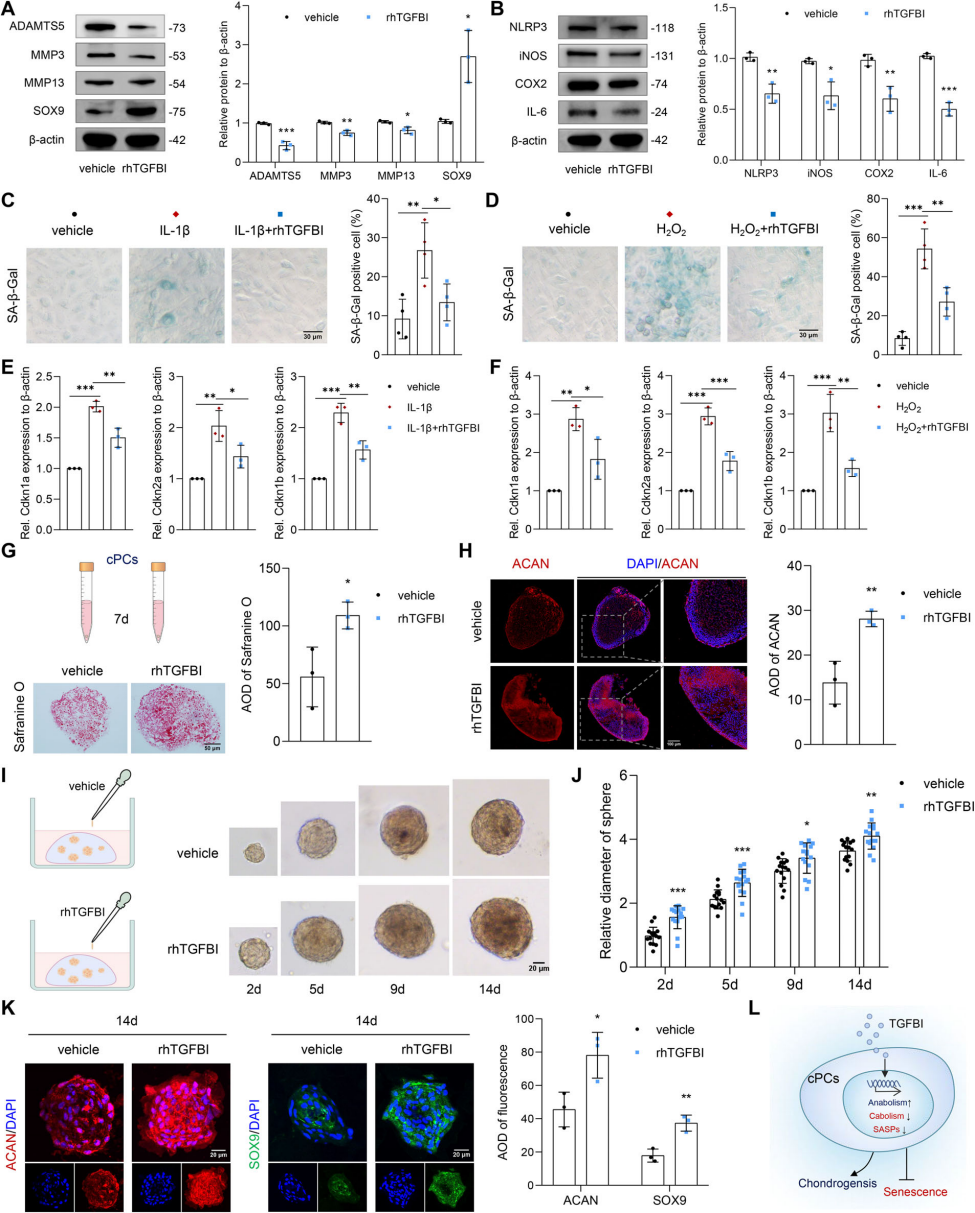
Recombinant human TGFBI enhances chondrogenesis and suppresses senescence in cPCs. (A, B) Western blot analysis of inflammatory SASP factors, catabolic and anabolic mediators in cPCs treated with or without rhTGFBI, unpaired t‐test, n = 3. (C, D) SA‐β‐galactosidase staining and quantitative analysis of cPCs under indicated treatments, one‐way ANOVA, n = 4. (E, F) qRT‐PCR quantitative analysis of *Cdkn1a*, *Cdkn2a*, and *Cdkn1b* mRNA levels in rat cPCs treated as indicated, one‐way ANOVA, n = 3. (G) Safranine O staining and quantification of cPC spheroids after chondrogenic induction in centrifuge tube pellet culture, unpaired t‐test, n = 3. (H) Immunofluorescence staining and quantification of ACAN in cPC spheroids after 14 days of chondrogenic induction, unpaired t‐test, n = 3. (I) Bright‐field 3D images of cPC spheroids cultured in Matrigel after chondrogenic induction for 2–14 days. (J) Quantification of spheroid diameter in Matrigel spheroid culture under indicated times, two‐way ANOVA, n = 15. (K) Immunofluorescence staining and quantification of anabolic biomarkers (ACAN, SOX9) in Matrigel‐cultured cPC spheroids, unpaired t‐test, n = 3. (L) Schematic diagram summarizing the role of TGFBI in regulating chondrogenesis and senescence in cPCs. Data are presented as mean ± SD; *p < 0.05, **p < 0.01, ***p < 0.001. SASP, senescence‐associated secretory phenotype; cPCs, condylar perichondrial cells.

Prompted by the robust protective phenotype observed in vivo, we aimed to clarify the direct effects of TGFBI on the putative progenitor cell population of the condyle, referred to as cPCs. We collected cPCs from the condylar perichondrium of rats or mice, followed by a series of enzymatic digestions and primary cultures (Figure S4A), and passages 1–2 of cPCs were used for subsequent ex vivo studies. The elevation of TGFBI in cPCs was confirmed by TGFBI immunoblot analysis upon rhTGFBI stimulation (Figure S4B). Treatment of isolated rat cPCs with rhTGFBI in vitro resulted in a potent anti‐catabolic and anti‐senescence effect. Western blot analysis showed that rhTGFBI significantly suppressed the protein levels of matrix‐degrading enzymes (ADAMTS5, MMP3, MMP13), key components of the inflammasome and inflammatory responses (NLRP3, iNOS, COX2), and the SASP factor IL‐6 (Figure 4A,B). Additionally, rhTGFBI increased the levels of the anabolic transcription factor SOX9 under basal conditions (Figure 4A). Consistently, the mRNA level of the cyclin‐dependent kinase inhibitor *Cdkn1b* was downregulated following rhTGFBI treatment (Figure S4C). Functionally, rhTGFBI effectively counteracted stress‐induced senescence in cPCs. Pre‐treatment with rhTGFBI markedly reduced the proportion of SA‐β‐Gal‐positive senescent cells induced by either the inflammatory cytokine IL‐1β or oxidative stress (H_2_O_2_) (Figure 4C,D). This anti‐senescence effect was further confirmed at the transcriptional level through qRT‐PCR, which showed suppressed levels of *Cdkn1a*, *Cdkn2a*, and *Cdkn1b* mRNA in these stress models (Figure 4E,F). Given the importance of integrin αV/β5 in mediating TGFBI signaling, we next examined the expression of integrin αV/β5 and αV/β3 in rat cPCs. Immunofluorescence staining demonstrated that cPCs majorly expressed integrin αV/β5 rather than αV/β3, with significant activation upon rhTGFBI treatment (Figure S4D, E). Furthermore, qRT‐PCR analysis revealed that rhTGFBI significantly downregulated the mRNA levels of catabolic markers (*Adamts5*, *Mmp13*, *Col10a1*) while up‐regulating anabolic markers (*Acan*, *Col2a1*, *Sox9*) in rat cPCs (Figure S4F, G).

In chondrogenic differentiation assays, rhTGFBI enhanced the matrix production of cPCs, as evidenced by intensified Safranine O staining (Figure 4G) and elevated ACAN immunofluorescence (Figure 4H) in cPC spheroids formed in centrifuge tube culture. In 3D Matrigel culture systems, rhTGFBI treatment for 2 to 14 days increased the size of cPC spheroids compared to the vehicle treatment group (Figure 4I,J). Furthermore, immunofluorescence staining revealed enhanced production of ACAN and SOX9 in rhTGFBI‐treated cPC spheroids formed in 3D Matrigel culture systems (Figure 4K). Collectively, these in vitro findings demonstrate that TGFBI exerts direct chondro‐promotive and anti‐senescence effects on cPCs, positioning it as a key regulator of this progenitor cell population in fibrocartilage (Figure 4L).

### 2.5 *Tgfbi* Deficiency Accelerates Cartilage Degeneration and Subchondral Bone Loss in MIA‐induced TMJOA Mice

**FIGURE 5:**
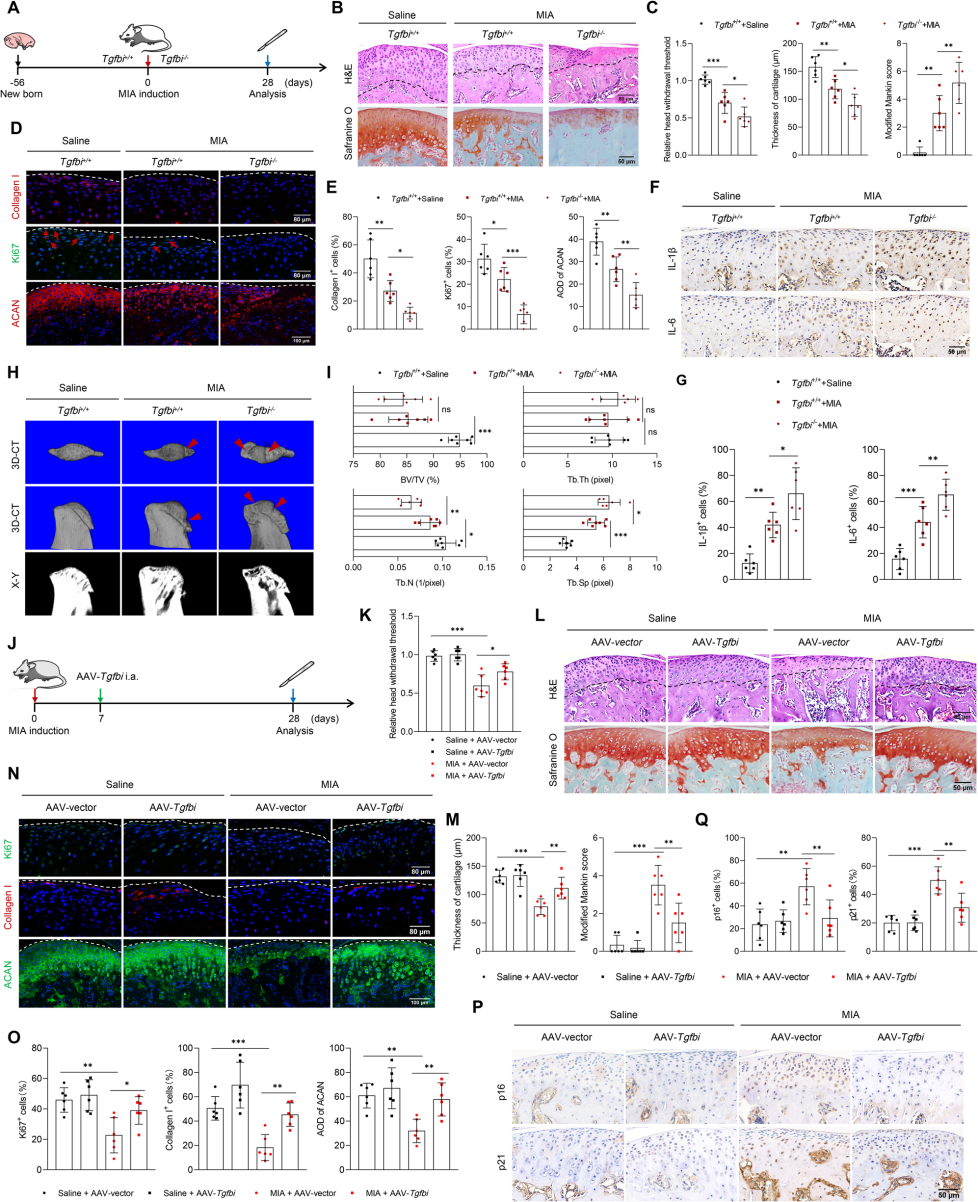
TGFBI mediates condylar cartilage degradation in MIA‐induced TMJOA mice. (A) Experimental timeline for analyzing TMJ tissues from MIA‐injected *Tgfbi* ^+/+^ and *Tgfbi* ^−/−^ mice. (B) H&E and Safranine O staining of condylar cartilage in MIA‐induced TMJOA mice. (C) Quantification of head withdrawal threshold, cartilage thickness and modified Mankin score from (B), one‐way ANOVA, n = 6. (D) Immunofluorescence staining of Collagen I, Ki67 and ACAN in mouse condylar cartilage. (E) Quantification analyzing the percentage of Collagen I‐positive, Ki67‐postive cells in condylar perichondrium, and AOD of ACAN within condylar cartilage from (D), one‐way ANOVA, n = 6. (F, G) Representative images of immunohistochemical staining for senescent biomarkers (IL‐1β, IL‐6) in mouse condylar cartilage, one‐way ANOVA, n = 6. (H, I) Representative micro‐CT images of mandibular condyle and quantitative analysis, one‐way ANOVA, n = 6. (J) Experimental timeline for AAV‐*Tgfbi* or AAV‐vector intra‐articular injection in MIA‐induced TMJOA mice. (K) Quantitative analysis of head withdrawal threshold at day 28, one‐way ANOVA, n = 6. (L) H&E and Safranine O staining of condylar cartilage from mice treated as indicated. (M) Quantitative analysis of cartilage thickness and modified Mankin score from (L), one‐way ANOVA, n = 6. (N) Immunofluorescence staining of Ki67, Collagen I and ACAN in the condylar cartilage of mice. (O) Quantification of Ki67‐positive and Collagen I‐positive cells in condylar perichondrium, and AOD of ACAN expression in condylar cartilage, one‐way ANOVA, n = 6. (P, Q) Immunohistochemical staining of senescent biomarkers (p16, p21) in mouse condylar cartilage, one‐way ANOVA, n = 6. Data are presented as mean ± SD; *p < 0.05, **p < 0.01, ***p < 0.001; ns, not significant. AOD, average optical density.

Next, we evaluated whether TGFBI modulates the joint response to chemical injury using the MIA‐induced TMJOA model (Figure 5A). *Tgfbi* ^−/−^ mice subjected to intra‐articular MIA injection displayed significantly more severe degenerative changes than MIA‐injected WT mice (Figure 5B). Von Frey tests indicated that the head withdrawal threshold further declined in *Tgfbi*‐deficient TMJOA mice compared to WT TMJOA mice (Figure 5C). Histological assessments via H&E and Safranine O staining revealed exacerbated condylar cartilage thinning, disorganization, chondrocyte loss, and proteoglycan depletion, resulting in significantly higher modified Mankin scores in the KO+MIA group (Figure 5B,C). Immunofluorescence analysis supported these findings, showing a more profound loss of ACAN matrix within the condylar cartilage of KO mice after intra‐articular MIA challenge (Figure 5D,E).

Alongside the loss of extracellular matrix, there was a greater reduction in Ki67‐positive proliferating chondrocytes and a decrease in Collagen I‐positive fibrochondrocytes in the condylar perichondrium of *Tgfbi*‐deficient TMJOA mice (Figure 5D,E). Simultaneously, the expression of inflammatory SASP cytokines (IL‐1β, IL‐6) was significantly more pronounced in the condylar cartilage of KO+MIA mice compared to the WT+MIA group (Figure 5F,G). Additional staining revealed elevated protein levels of the cell cycle arrest markers (p21 and p16), along with key catabolic enzymes (ADAMTS5, MMP13) in the condylar cartilage of *Tgfbi*‐deficient mice post‐MIA induction (Figure S5A, B). µCT analysis demonstrated that *Tgfbi* deficiency synergized with MIA to exacerbate subchondral bone destruction, leading to further reductions in Tb. N and increased Tb. Sp of bone, compared to WT mice receiving MIA (Figure 5H,I). These results establish that TGFBI is not only crucial for maintaining chondrocyte homeostasis but also plays a critical protective role in mitigating the progression of MIA‐induced TMJOA.

### 2.6 Intra‐articular TGFBI Overexpression Ameliorates TMJOA Progression in Mice

To assess the therapeutic potential of TGFBI supplementation in TMJOA mice, we performed intra‐articular delivery of adeno‐associated virus carrying *Tgfbi* (AAV‐*Tgfbi*) into the mouse TMJs one week after MIA induction (Figure 5J). This intervention significantly alleviated joint pain in MIA‐induced TMJOA mice, which exhibited an enhanced head withdrawal threshold following intra‐articular AAV‐*Tgfbi* injection (Figure 5K). Histological analysis demonstrated that AAV‐*Tgfbi* treatment improved condylar cartilage structure, increased cartilage thickness, enhanced Safranine O staining, and lowered the modified Mankin score compared to MIA‐injected mice receiving a control AAV‐vector (Figure 5L,M). The successful overexpression of TGFBI in the condylar cartilage was confirmed by immunofluorescence staining, which showed significantly increased TGFBI fluorescence intensity in the AAV‐*Tgfbi* group compared to the AAV‐vector group of TMJOA mice (Figure S5C, D). Immunofluorescence staining revealed that TGFBI overexpression enhanced the deposition of ACAN matrix and increased the proportion of Collagen I‐positive fibrochondrocytes in the perichondrium region of the condylar cartilage (Figure 5N,O).

Furthermore, intra‐articular administration of AAV‐*Tgfbi* promoted cellular proliferation, as indicated by an increased proportion of Ki67‐positive chondrocytes in condylar perichondrium (Figure 5N,O), and reduced the expression of cellular senescence markers p21 and p16 in the mouse condylar cartilage (Figure 5P,Q). It also abolished the MIA‐induced upregulation of catabolic enzymes (ADAMTS5, MMP13) and inflammatory SASP factors (IL‐1β, IL‐6) within the condylar cartilage of TMJOA mice (Figure S5E, F). The protective effects extended to the subchondral bone, where µCT analysis confirmed that AAV‐*Tgfbi* administration mitigated MIA‐induced subchondral bone destruction in TMJOA mice, significantly improving parameters such as BV/TV and Tb. N, while reducing Tb. Sp (Figure S5G, H). These results provide compelling proof‐of‐concept that restoring TGFBI expression in osteoarthritic fibrocartilage can effectively halt the progression of degenerative joint disease and promote a reparative environment.

### 2.7 TGFBI Negatively Regulates Protein O‐GlcNAcylation in Osteoarthritic cPCs

**FIGURE 6:**
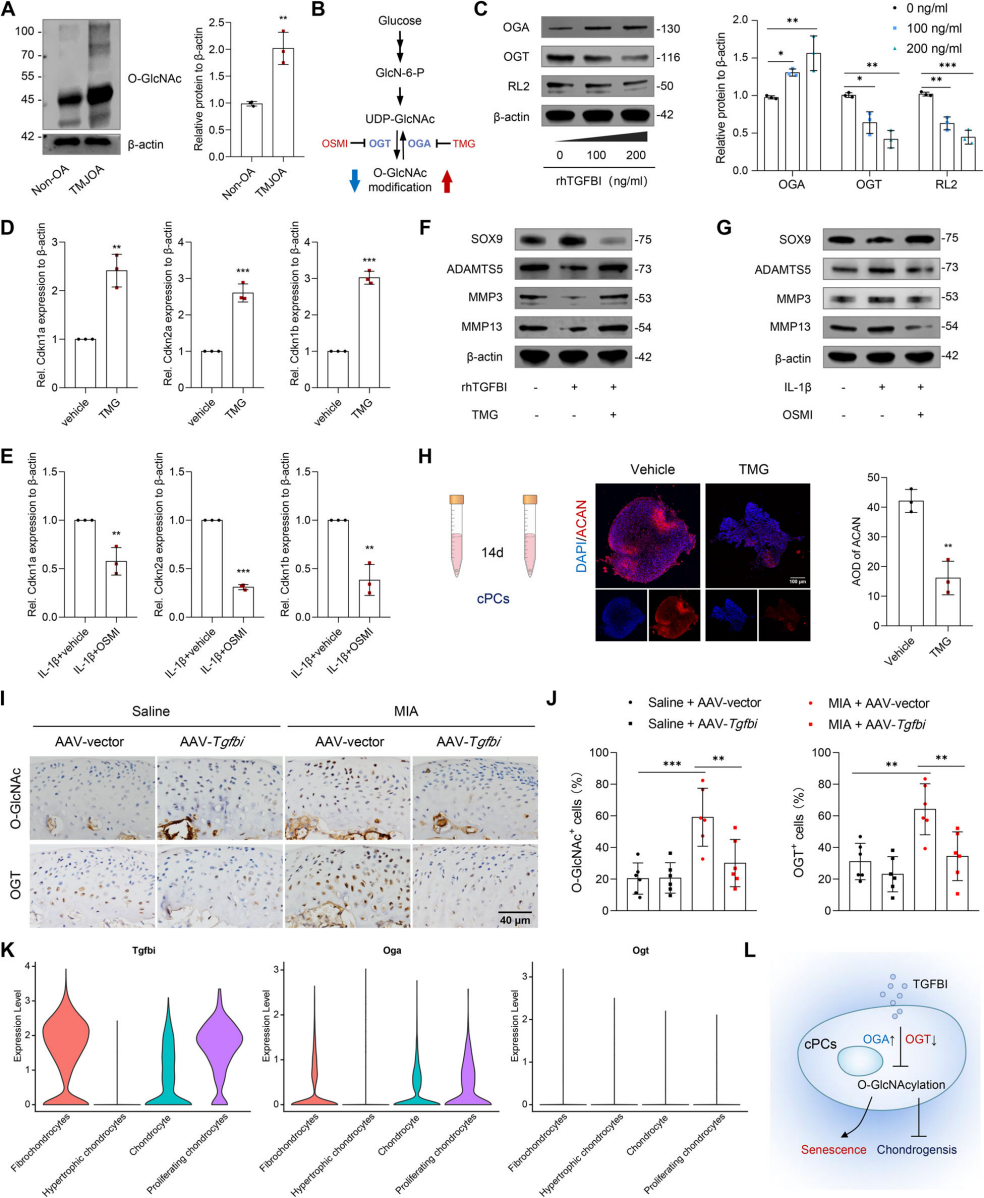
TGFBI suppresses O‐GlcNAcylation in osteoarthritic condylar perichondrial cells. (A) Western blot analysis of global O‐GlcNAcylation (RL2) in synovial tissues from TMJOA and non‐OA patients, unpaired t‐test, n = 3. (B) The compounds TMG and OSMI used in the present study targeting O‐GlcNAc modification. (C) Western blot analysis of OGA, OGT, and O‐GlcNAc levels in rat cPCs treated with different doses of rhTGFBI, one‐way ANOVA, n = 3. (D, E) qRT‐PCR analysis of senescence‐related mRNA levels (*Cdkn1a*, *Cdkn2a*, *Cdkn1b*) in rat cPCs, unpaired t‐test, n = 3. (F, G) Western blot analysis of catabolic and anabolic factors in cPCs under indicated treatments. (H) Immunofluorescent staining and quantification of ACAN expression in cPC spheroids treated with TMG for 14 days, unpaired t‐test, n = 3. (I, J) Immunohistochemical staining (I) and quantification (J) of O‐GlcNAc and OGT in condylar cartilage from TMJOA mice, with or without AAV‐*Tgfbi* treatment, one‐way ANOVA, n = 6. (K) Violin plots showing expression levels of *Tgfbi*, *Oga*, and *Ogt* across four chondrocyte subpopulations. (L) Schematic illustrating TGFBI‐mediated inhibition of O‐GlcNAcylation in regulating cPC chondrogenesis and senescence. Data are presented as mean ± SD; *p < 0.05, **p < 0.01, ***p < 0.001. TMG, Thiamet G; OSMI, OSMI‐1; cPCs, condylar perichondrial cells.

Analysis of our scRNA‐seq data indicated that *Tgfbi* is primarily expressed in proliferating chondrocytes and fibrochondrocytes (Figure 6K). Notably, both *Tgfbi* and *Oga* (a key enzyme that removes O‐GlcNAcase to reduce protein O‐GlcNAcylation) exhibited low expression in the hypertrophic chondrocyte subtype (Figure 6K), suggesting a potential synergistic protective role of *Tgfbi* and *Oga* in fibrocartilage. Additionally, the expression of *Oga* and the cartilage extracellular matrix gene *Col2a1* was reduced in chondrocytes from KO mice compared to WT mice (Figure S6A, B). Given the similar alterations observed with OGA, we hypothesized that TGFBI might exert its effects by modulating this critical post‐translational modification, O‐GlcNAcylation. Immunohistochemical staining exhibited the levels of global protein O‐GlcNAcylation (detected by the RL2 antibody) were increased in the damaged area of human TMJOA condylar cartilage, compared with undamaged area (Figure S6C). Analysis of human synovial tissues revealed significantly elevated global O‐GlcNAcylation levels in TMJOA samples compared to non‐OA controls (Figure 6A). These observations led us further to investigate a potential link between TGFBI and this nutrient‐sensitive modification in and ex vivo.

In vitro, treatment of cPCs with rhTGFBI resulted in a dose‐dependent reduction in global O‐GlcNAc levels (Figure 6C). Consistently, qRT‐PCR analysis showed that rhTGFBI treatment significantly upregulated *Oga* mRNA expression while downregulating *Ogt* mRNA levels in rat cPCs (Figure S6H), further supporting the regulatory effect of TGFBI on the O‐GlcNAcylation enzymatic machinery at the transcriptional level. Mechanistically, this phenomenon was associated with a decrease in the protein levels of the enzyme responsible for the addition of O‐GlcNAc (O‐GlcNAc transferase, OGT), alongside a corresponding increase in the levels of the enzyme responsible for its removal (O‐GlcNAcase, OGA) (Figure 6B,C). Functionally, pharmacologically elevating O‐GlcNAcylation with Thiamet G (TMG, an OGA inhibitor) in cPCs promoted a catabolic and senescent state, upregulating the expression of senescent factors (*Cdkn1a*, *Cdkn2a*, *Cdkn1b*) and degenerative markers (*Adamts5*, *Mmp13*, *Col10a1*) (Figure 6D, Figure S6D). Conversely, inhibiting O‐GlcNAcylation with OSMI‐1 (OSMI, an OGT inhibitor) suppressed the expression of these senescent factors and upregulated anabolic factors (*Acan*, *Col2a1*, *Sox9*) under corresponding conditions (Figure 6E, Figure S6E). Importantly, the modulatory effects of TGFBI are dependent on O‐GlcNAcylation status: TMG treatment reversed the pro‐anabolic and catabolic‐inhibiting effects of rhTGFBI incubation, while OSMI‐1 treatment blocked the pro‐catabolic and anabolic‐suppressing effects induced by IL‐1β stimulation (Figure 6F,G). Furthermore, elevated O‐GlcNAcylation impaired the chondrogenic differentiation of cPC spheroids, reducing spheroid size and matrix ACAN expression in centrifuge tube culture model (Figure 6H).

In vivo, immunohistochemistry confirmed a significant increase in O‐GlcNAc and OGT levels in the condylar cartilage of MIA‐induced TMJOA mice, which was effectively normalized by AAV‐*Tgfbi* treatment (Figure 6I,J). Conversely, *Tgfbi* deficiency further elevated O‐GlcNAc and OGT protein levels in the condylar cartilage of MIA‐induced TMJOA mice (Figure S6F,G). These data establish that TGFBI functions as a physiological suppressor on protein O‐GlcNAcylation in cPCs, and this regulation is integral to its chondro‐protective and anti‐senescence functions (Figure 6L).

### 2.8 O‐GlcNAcylation Promotes the Transcriptional Activation of *Adamts16* in cPCs

**FIGURE 7:**
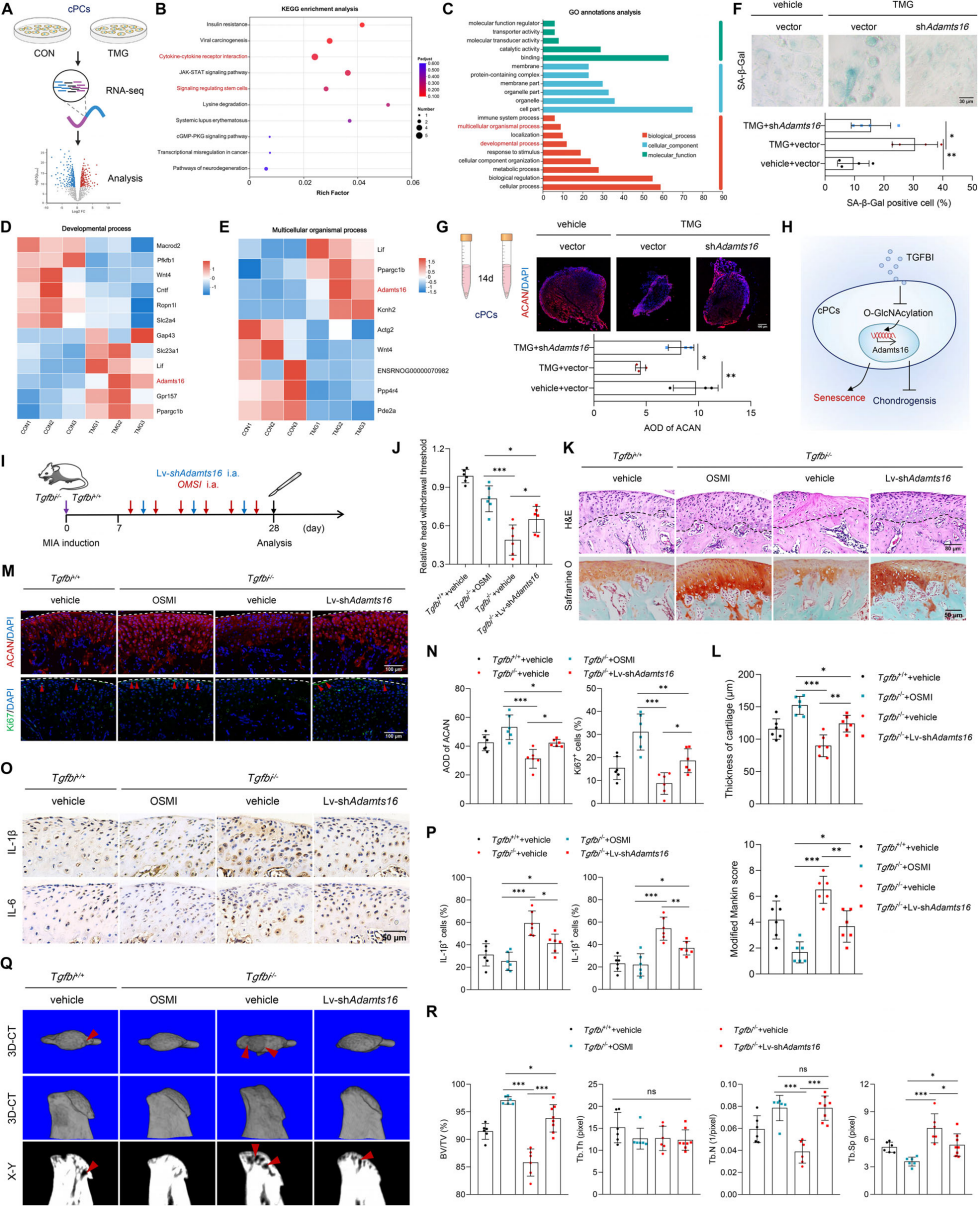
O‐GlcNAcylation promotes transcriptional activation of *Adamts16* in cPCs. (A) Workflow for bulk RNA‐sequencing of cPCs treated with or without TMG. (B, C) KEGG pathway enrichment (B) and GO term annotation (C) of DEGs. (D, E) Heatmaps of DEGs associated with developmental process (D) and multicellular organismal process (E). (F) SA‐β‐galactosidase staining and quantification of mouse cPCs under indicated treatments, one‐way ANOVA, n = 4. (G) Immunofluorescence staining and quantification of ACAN in mouse cPC spheroids after indicated treatments for 14 days, one‐way ANOVA, n = 3. (H) Schematic summarizing the role of O‐GlcNAcylation in activating *Adamts16* transcription and mediating cPCs chondrogenesis and senescence. (I) Experimental timeline for intra‐articular treatments with Lv‐sh*Adamts16* or OSMI‐1 in MIA‐injected *Tgfbi* ^−/−^ mice. (J) Quantitative analysis of head withdrawal threshold at day 28, one‐way ANOVA, n = 6. (K) H&E and Safranine O staining of condylar cartilage from TMJOA mice. (L) Quantification of cartilage thickness and modified Mankin score from (K), one‐way ANOVA, n = 6. (M) Immunofluorescence staining of ACAN and Ki67 in mouse condylar cartilage. (N) Quantification of ACAN expression in condylar cartilage, and Ki67‐positive cells in condylar perichondrium from (M), one‐way ANOVA, n = 6. (O, P) Immunohistochemical staining and quantification of senescent biomarkers (IL‐1β, IL‐6) in mouse condylar cartilage, one‐way ANOVA, n = 6. (Q, R) Representative micro‐CT images of mandibular condyle and quantitative analysis, one‐way ANOVA, n = 6. Data are presented as mean ± SD; *p < 0.05, **p < 0.01, ***p < 0.001; ns, not significant. TMG, Thiamet G; OSMI, OSMI‐1; cPCs, condylar perichondrial cells; DEGs, differentially expressed genes; AOD, average optical density.

To identify the downstream effector of O‐GlcNAcylation in cPCs, we conducted bulk RNA sequencing on vehicle‐ or TMG‐treated rat cPCs (Figure 7A). Quality control and differential expression analysis confirmed high data quality and distinct separation between the two groups (Figure S7A–D). We identified 137 differentially expressed genes (DEGs, |log2FC|≧1, p‐value<0.05), including 57 up‐regulated genes and 80 down‐regulated genes (Figure S7E–G). KEGG and GO enrichment analyses revealed significant changes in pathways related to “cytokine‐cytokine receptor interaction” and “signaling regulating stem cells”, and in biological processes including “multicellular organismal process” and “developmental process” (Figure 7B,C). Among the upregulated DEGs, *Adamts16*, a member of the ADAMTS protease family, was notably induced by TMG treatment and was associated with both “multicellular organismal process” and “developmental process” (Figure 7D,E).

We then validated the functional relevance of *Adamts16* in mouse cPCs using lentivirus‐mediated knockdown. Lentivirus‐mediated shRNA knockdown of *Adamts16* (Lv‐sh*Adamts16*) effectively reduced *Adamts16* expression at both mRNA and protein levels (Figure S7H). Importantly, *Adamts16* knockdown significantly reduced the proportion of SA‐β‐Gal‐positive senescent cells induced by TMG treatment in mouse cPCs (Figure 7F). Additionally, qRT‐PCR analysis showed that *Adamts16* knockdown decreased the expression of senescence markers (*Cdkn1a*, *Cdkn2a*, *Cdkn1b*) and catabolic factors (*Adamts5*, *Mmp13*) in IL‐1β‐induced mouse cPCs (Figure S7I, J). In chondrogenic pellet cultures of cPCs, silencing *Adamts16* partially rescued the impairment in matrix production (ACAN expression) and the size of cPC spheroids caused by elevated O‐GlcNAcylation (Figure 7G). These findings indicate that TGFBI promotes chondrogenesis and exhibits anti‐senescence effects on cPCs, potentially by inhibiting O‐GlcNAcylation‐mediated transcriptional activation of *Adamts16* (Figure 7H).

### 2.9 Pharmacological Inhibition of O‐GlcNAcylation Provides Superior Therapeutic Efficacy to *Adamts16* Knockdown in *Tgfbi*‐Deficient TMJOA Mice

We then explored two therapeutic strategies in the context of *Tgfbi*‐deficient TMJOA: global inhibition of O‐GlcNAcylation and targeted knockdown of its downstream effector, *Adamts16*. MIA‐injected *Tgfbi* KO mice received intra‐articular injections of either the OGT inhibitor OSMI‐1 or Lv‐sh*Adamts16* (Figure 7I). Von Ferry tests indicated that both O‐GlcNAcylation inhibition and *Adamts16* knockdown alleviated joint pain by increasing the head withdrawal threshold in *Tgfbi*‐deficient TMJOA mice (Figure 7J). Furthermore, both treatments resulted in significant histological improvements compared to vehicle‐treated *Tgfbi*‐deficient TMJOA mice, marked by increased cartilage thickness, enhanced safranine O staining, and reduced modified Mankin scores (Figure 7K,L). Both interventions also elevated the expression of the anabolic marker ACAN in the condylar cartilage, increased the number of Ki67‐positive proliferating chondrocytes and Collagen I‐positive fibrochondrocytes in the condylar perichondrium (Figure 7M,N, Figure S8A,B). Immunohistochemical staining revealed reduced levels of SASP factors (IL‐1β, IL‐6, p21, p16) and catabolic enzymes (ADAMTS5, MMP13) in both OSMI‐1‐treated and Lv‐sh*Adamts16*‐treated TMJOA mice compared to vehicle‐treated counterparts (Figure 7O,P, Figure S8C, D). Furthermore, both treatments improved condylar subchondral bone integrity, enhancing BV/TV and Tb. N while decreasing Tb. Sp of bone in OSMI‐1‐treated and Lv‐sh*Adamts16*‐treated *Tgfbi*‐deficient TMJOA mice (Figure 7Q,R).

Notably, direct inhibition of O‐GlcNAcylation with OSMI‐1 demonstrated superior therapeutic efficacy across nearly all measured parameters—including joint pain, articular cartilage histology, metabolic/senescent biomarker expression, and bone micro‐architecture—compared to *Adamts16* knockdown alone (Figure 7J,R, Figure S8A–D). Moreover, OSMI‐1 treatments rather than Lv‐sh*Adamts16* injections, effectively reduced the elevated O‐GlcNAcylation levels and OGT expression in the condylar cartilage of *Tgfbi*‐deficient TMJOA mice (Figure S8E, F). This confirms that the therapeutic benefits of these interventions are mediated by the suppression of the O‐GlcNAcylation pathway. Collectively, these findings further highlight that while ADAMTS16 is a significant effector, O‐GlcNAcylation governs broader downstream signals of TGFBI.

### 2.10 O‐GlcNAcylation Stabilizes the ADAMTS16 Protein via Direct Interaction With OGT

**FIGURE 8:**
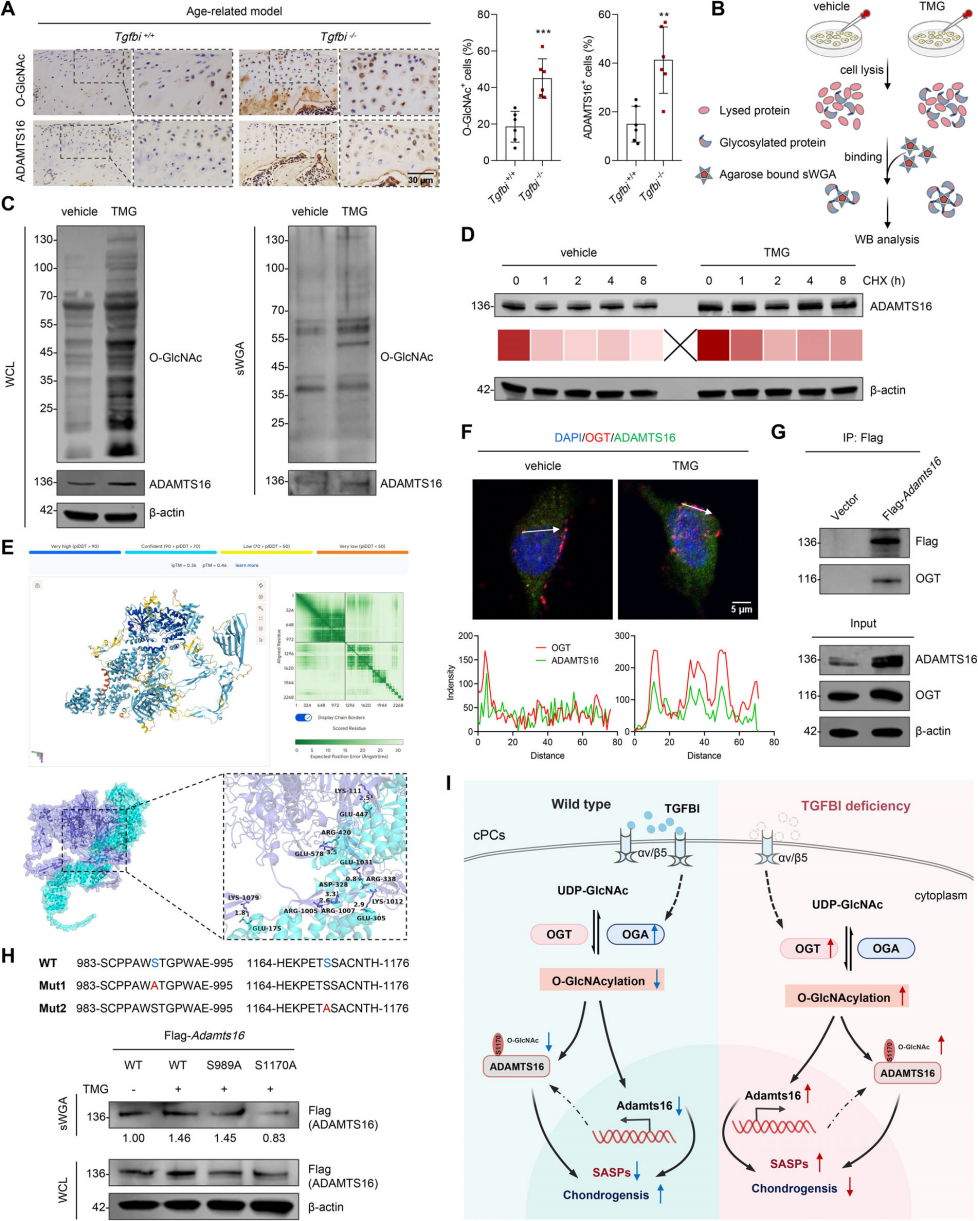
O‐GlcNAcylation stabilizes ADAMTS16 protein via direct interaction with OGT. (A) Immunohistochemical staining and quantification of O‐GlcNAc and ADAMTS16 in condylar cartilage from age‐related mice, unpaired t‐test, n = 6. (B) Workflow for sWGA pull‐down assay in TMG‐treated ATDC5 chondrogenic cells. (C) Western blot analysis of O‐GlcNAcylated proteins after sWGA pull‐down, cells treated with or without TMG. (D) CHX chase assay assessing ADAMTS16 protein stability in ATDC5 chondrogenic cells after treated with TMG. (E) Predicted interaction model between mouse ADAMTS16 and OGT generated by AlphaFold3, and protein‐protein docking performed by Docking Web Server (GRAMM). (F) Immunofluorescence co‐staining and co‐localization analysis of OGT and ADAMTS16 in ATDC5 chondrogenic cells. (G) Co‐immunoprecipitation assay verifying the interaction between OGT and ADAMTS16 in HEK‐293T cells, transfected with Flag‐vector or Flag‐tagged Adamts16. (H) sWGA pull‐down assay and quantification in HEK‐293T cells overexpressing wild‐type or mutant *Adamts16* (S989A, S1170A). (I) Schematic model of TGFBI regulating cPCs homeostasis via O‐GlcNAcylation‐dependent ADAMTS16 transcriptional activation and protein stabilization. Data are presented as mean ± SD; **p < 0.01, ***p < 0.001. TMG, Thiamet G; sWGA, succinylated wheat germ agglutinin; WCL, whole cell lysate; CHX, cycloheximide.

Given that O‐GlcNAcylation inhibition exhibited broader effects than genetic *Adamts16* knockdown on fibrocartilage degeneration in TMJOA mice, we hypothesized that O‐GlcNAcylation may also regulate the post‐translational modification of ADAMTS16, in addition to its transcriptional activation. We investigated the mechanism by which O‐GlcNAcylation mediates the stabilization of the ADAMTS16 protein through post‐translational modification. Immunostaining revealed elevated levels of ADAMTS16 protein in the condylar cartilage of age‐related *Tgfbi* ^−/−^ mice, which correlated with higher O‐GlcNAc signals (Figure 8A). Moreover, *Tgfbi* deficiency increased the protein level of ADAMTS16 in the condylar cartilage of MIA‐induced TMJOA mice (Figure S9A, B). In vitro, succinylated wheat germ agglutinin (sWGA) pull‐down assays in TMG‐treated ATDC5 chondrogenic cells confirmed that ADAMTS16 is an O‐GlcNAcylated protein, demonstrating elevated levels of both total and O‐GlcNAcylated protein (Figure 8B,C). Cycloheximide (CHX) chase assays indicated that TMG treatment promoting O‐GlcNAcylation significantly prolonged the half‐life of the ADAMTS16 protein, suggesting that O‐GlcNAcylation modification enhances its stability (Figure 8D).

In silico molecular docking using AlphaFold3 predicted a potential direct interaction between mouse ADAMTS16 and OGT (Figure 8E). Protein‐protein interaction modeling generated by PyMOL predicted multiple potential binding sites between mouse ADAMTS16 and mouse OGT, such as LYS1079 of ADAMTS16 (Q69Z28) and GLU175 of OGT (Q8CGY8) (Figure 8E). Furtherly, immunofluorescence double staining confirmed the co‐localization of OGT and ADAMTS16 in ATDC5 chondrogenic cells (Figure 8F). The direct interaction of OGT and ADAMTS16 protein was further validated by co‐immunoprecipitation assay transfecting Flag‐tagged *Adamts16* or Flag‐vector to HEK‐293T cells (Figure 8G). Bioinformatic prediction (NetOGlyc‐4.0) and cross‐species alignment identified two conserved putative O‐GlcNAcylation modification sites on mouse ADAMTS16: Ser989 and Ser1170 (Figure S9C, D). Site‐directed mutagenesis (S989A, S1170A) of *Adamts16* and subsequent sWGA pull‐down assays revealed that mutation of Ser1170, but not Ser989, significantly reduced the O‐GlcNAcylation level of ADAMTS16 protein (Figure S9E, F, Figure 8H), identifying Ser1170 as a critical O‐GlcNAcylation modification site influencing its protein stability. These findings establish a direct mechanistic link: *Tgfbi* deficiency elevates O‐GlcNAcylation, promoting the transcriptional activation of *Adamts16* and OGT‐mediated post‐translational modification of ADAMTS16 at Ser1170. This leads to its stabilization and accumulation, driving chondrocyte metabolic disorders and senescent phenotypes, ultimately resulting in fibrocartilage dysfunction and TMJOA pathogenesis (Figure 8I).

## 3 Discussion

This study systematically identifies a novel and crucial role for TGFBI in maintaining the homeostasis of the temporomandibular joint, especially within its distinct fibrocartilage compartment. We provide converging evidence from patient samples, TMJOA models, a genetic mouse model, and in vitro cellular studies to establish that TGFBI functions as a critical protective factor against TMJOA pathogenesis. The core mechanism of this protection lies in TGFBI's ability to suppress global protein O‐GlcNAcylation within cPCs, which in turn restrains the expression and stabilization of the matrix‐degrading enzyme ADAMTS16. Our findings position the TGFBI/O‐GlcNAcylation/ADAMTS16 axis as a vital regulatory node in maintaining TMJ fibrocartilage health and as a promising target for disease‐modifying therapy.

First, our clinical data reveal reduced TGFBI levels in the synovial fluid and damaged cartilage of TMJOA patients, establishing the direct relevance of TGFBI to human disease. This downregulation correlates with the severity of condylar degeneration, suggesting that the loss of TGFBI may serve as both a biomarker and a contributing factor in TMJOA progression. Additionally, the prominent expression of TGFBI in the superficial perichondrial layers of the condyle aligns with its hypothesized role in regulating the progenitor cell niche, which is critical for fibrocartilage maintenance and repair [14, 15]. Furthermore, the phenotype observed in our *Tgfbi* knockout mice strongly corroborates this hypothesis, consistent with prior reports indicating that *Tgfbi* deficiency results in delayed skeletal development [27]. We observed a delay in postnatal cartilage development and an exacerbation of age‐related and MIA‐induced TMJOA phenotype in KO mice. This finding demonstrates that TGFBI is essential for both developmental morphogenesis and long‐term homeostasis of the TMJ. The shift toward a hypertrophic chondrocyte fate, revealed by scRNA‐seq, provides a cellular explanation for the degenerative changes of condylar cartilage in KO mice. Hypertrophy represents a terminal differentiation step in endochondral ossification and is a hallmark of OA pathology, characterized by increased matrix mineralization, expression of collagen X, and elevated proteolytic activity [46, 47]. *Tgfbi* deficiency seems to disrupt the balanced differentiation of cPCs, leading to the activation of a harmful hypertrophic pathway.

The finding that TGFBI's chondroprotective effect is mediated by the inhibition of protein O‐GlcNAcylation represents a significant advance in our understanding of cartilage biology. O‐GlcNAcylation, which is a nutrient‐sensitive post‐translational modification, has been identified as a crucial regulator of cellular stress responses, transcription, and protein stability [41, 48, 49]. Its dysregulation is implicated in various age‐related diseases, including diabetes, neurodegenerative diseases, and OA [50, 51]. We found elevated O‐GlcNAc levels in human TMJOA specimen and in *Tgfbi*‐deficient murine TMJOA condylar cartilage, suggesting a potential pathogenic link. TGFBI appears to act as a brake on this modification in cPCs, pharmacologically elevating O‐GlcNAcylation mimicked the senescent and catabolic phenotype observed with *Tgfbi* deficiency. Conversely, inhibiting O‐GlcNAcylation with OSMI‐1 proved to be therapeutic, even in the deficiency of *Tgfbi*. Furthermore, Kang et al. discovered that pharmacological modulation of O‐GlcNAcylation regulates GATA4 stability and influences post‐traumatic OA in mice [41]. These findings position TGFBI upstream in a critical metabolic‐sensing pathway within cartilage cells. The functional pleiotropy of TGFBI in different tissues is largely dictated by its interaction with distinct integrin receptors. A recent landmark study by Bi et al. demonstrated that integrin αV/β5 serves as a pivotal receptor in fibrocartilage and mediates fibrocartilaginous transformation during OA progression [43]. Our clinical and single‐cell data corroborate this finding, showing that integrin αV/β5 is the predominant receptor expressed on condylar perichondrial cells and that its expression correlates positively with TGFBI levels.

Our integrated transcriptomic and biochemical analyses revealed that ADAMTS16 acts as a key downstream effector of O‐GlcNAcylation in this context. As a member of the ADAMTS family of proteases, ADAMTS16 is recognized for its role in ECM remodeling [52, 53]. Our study shows that O‐GlcNAcylation, potentiated by the loss of *Tgfbi*, drives both the transcriptional upregulation and, more importantly, the post‐translational stabilization of the ADAMTS16 protein. CHX chase and sWGA pull‐down experiments provide direct evidence that O‐GlcNAc modification prolongs ADAMTS16 half‐life. The predicted and validated interaction with OGT, along with the identification of Ser1170 as a key O‐GlcNAcylation site, support a model where OGT directly modifies ADAMTS16, increasing its stability and activity. This dual regulation—transcriptional and post‐translational—clarifies the significant impact of O‐GlcNAcylation on ADAMTS16 abundance. Members of the ADAMTS family are classically known for their aggrecanase activity and cleavage of proteoglycans. Previous study has identified *Adamts16* as one of the most significantly upregulated genes in OA synovium and cartilage compared to the fractured tissues [54]. This suggests its potential role in causing the degradation of extracellular matrix and triggering the pathogenesis of osteoarthritis. How its proteolytic activity mechanistically triggers the hypertrophic shift and matrix breakdown in the TMJ fibrocartilage, require systematic substrate‐trapping assays and degradomic profiling. Although *Adamts16* knockdown showed benefits, the greater efficacy of global O‐GlcNAcylation inhibition suggests that other O‐GlcNAcylated proteins likely contribute to the OA phenotype, indicating a broader therapeutic target.

The therapeutic implications of our findings are substantial. The successful treatment of TMJOA in mice through intra‐articular AAV‐mediated TGFBI overexpression validates its potential as a biologic agent for genetic therapy. Even more compelling is the effectiveness of the small‐molecule OGT inhibitor OSMI‐1, pharmacological inhibition of O‐GlcNAcylation not only rescued the OA phenotype in *Tgfbi*‐deficient mice but also surpassed results of targeted *Adamts16* knockdown. This indicates that modulating this essential metabolic pathway, O‐GlcNAcylation modification, presents a powerful strategy to intercept TMJOA progression, regardless of various initiating factors. Targeting O‐GlcNAcylation could potentially normalize the dysregulated chondrocyte differentiation, senescence, and metabolic activity.

Several questions remain for future investigation. The exact signaling pathway by which extracellular TGFBI influences intracellular OGT activity or expression remains unknown. It is essential to identify potential receptors or co‐factors that mediate TGFBI's action on cPCs. Additionally, the specific ECM substrates of ADAMTS16 in TMJ fibrocartilage and its precise mechanism in driving hypertrophy and degradation require detailed proteomic analysis. Exploring the potential crosstalk between the TGFBI pathway and other critical signaling networks in OA, such as Wnt or TGF‐β/BMP signaling, is also warranted.

In conclusion, this study reveals a previously unrecognized mechanism by which TGFBI protects TMJ integrity by continually suppressing protein O‐GlcNAcylation in progenitor‐like cPCs. The loss of *Tgfbi*, as observed in TMJOA, unleashes O‐GlcNAcylation, which subsequently stabilizes and upregulates the protease ADAMTS16, leading to chondrocyte hypertrophy, senescence, and matrix destruction. This work redefines TGFBI from merely an ECM component to a dynamic regulator of chondrocyte metabolism and fate. It proposes both TGFBI supplementation and, more effectively, O‐GlcNAcylation inhibition as novel therapeutic strategies for TMJOA, a debilitating condition with limited treatment options.

## 4 Materials and Methods

### 4.1 Clinical Samples Collection

Clinical research involving human samples received approval from the Ethics Committee of the School and Hospital of Stomatology, Wuhan University (Approval No. 2021‐A28). In this study, TMJ synovial fluid samples were collected from TMD patients. These patients were diagnosed with degenerative joint disease (TMJOA group) or subluxation (non‐OA controls) based on the Diagnostic Criteria for Temporomandibular Disorders (DC/TMD) [55]. The degree of condylar subchondral bone degeneration was assessed using a CBCT‐based staging system that evaluates human condylar shape and height, as referenced in previous research (Table S1) [56]. Condylar cartilage and synovial tissues were collected from patients who underwent arthroplasty for condylar fractures or TMJOA, following the acquisition of written informed consent.

### 4.2 Animals

All animal procedures were approved by the Laboratory Animal Ethics Committee of the Hospital of Stomatology at Wuhan University (Approval No. S0792302043). These procedures were conducted following the National Research Council's Guide for the Care and Use of Laboratory Animals and in compliance with ARRIVE 2.0 guidelines.

Male WT C57BL/6 mice and Sprague–Dawley (SD) rats, used for animal models and in vitro experiments, were obtained from the Experimental Animal Center of Hubei Province. *Tgfbi* knockout (KO) mice on a C57BL/6 background were generated by Suzhou Cyagen Biosciences (strain S‐KO‐05374). The target region selected for the knockout was exons 3–7 of the *Tgfbi* gene located on mouse chromosome 13. To generate the knockout mice, Cas9 mRNA and guide RNAs were co‐injected into fertilized eggs. Genotyping was then performed using PCR followed by Sanger sequencing, which confirmed a 5,351‐bp deletion in the targeted region.

### 4.3 TMJOA Models

Experiments were performed using monosodium iodoacetate (MIA)‐induced and age‐related TMJOA models. For the MIA‐induced model, mice received an intra‐articular injection of 0.4 mg MIA (Sigma‐Aldrich, #57858) dissolved in 20 µL saline into the anterosuperior compartment of bilateral TMJs [57]. Mice in the control group received an equal volume of isotonic saline. For the age‐related degeneration model, 12‐month‐old male mice were used to assess age‐associated joint degeneration [44, 45, 58].

### 4.4 Intra‐Articular Injection of Virus or Pharmacological Agents

For TGFBI overexpression, adeno‐associated virus carrying *Tgfbi* (AAV‐*Tgfbi*) or empty vector (AAV‐vector) (Genechem Co., Ltd.) was microinjected (1.0×10^1^ ^2^ v.g./mL, 10 µL) into the TMJ cavity one week after MIA induction.

For *Adamts16* knockdown, lentiviral vectors expressing shRNA against *Adamts16* (Lv‐sh*Adamts16*) or a nontargeting control (Lv‐vector) (Genechem Co., Ltd.) were injected (1.0×10^8^ TU/mL, 10 µL) weekly for three weeks, starting one week after MIA injection.

To inhibit O‐GlcNAcylation, the O‐GlcNAc transferase (OGT) inhibitor OSMI‐1 (MCE, HY‐119738) was administered via intra‐articular injection (10 nmol in 10 µL) twice a week for three weeks, starting one week after MIA induction.

### 4.5 Electronic Von Frey Tests

Mice were acclimatized in transparent testing chambers with a wire mesh floor prior to testing. The probe of the electronic von Frey was applied perpendicularly to the skin overlying the TMJ, located approximately 3–4 mm anterior and inferior to the external auditory meatus. A gradual force was applied until a withdrawal response—such as a sharp head flick, face wiping, or vocalization—was elicited. The force at which this response occurred was recorded as the head withdrawal threshold. Testing was conducted bilaterally in a blinded manner and repeated 5 times, with a minimum interval of 30 s between stimulations.

### 4.6 Alcian Blue and Alizarin Red Staining of Fetal Mice

Newborn mice skeletal staining with Alcian blue and Alizarin red was performed as previously described [59]. In summary, neonatal mice were euthanized, eviscerated, and preserved in 95% ethanol. Following the excision of the dermal layer, specimens were stained with a 15% Alcian blue solution (Sigma‐Aldrich) to visualize cartilage, which was subsequently followed by overnight staining with a 12% Alizarin red solution (Sigma‐Aldrich) to identify mineralized tissues. The samples were then cleared using a solution composed of 1% KOH and 20% glycerol until the soft tissues became transparent, after which they were transferred to increasing concentrations of glycerol and stored in a 50% glycerol solution.

### 4.7 Single‐Cell Dissociation

Condylar cartilage tissues were dissected from 8‐week‐old WT and *Tgfbi* ^−/−^ adult mice, with 8 condylar cartilages pooled into a single sample per group. The tissues were minced on ice and digested in PBS with 0.2% collagenase type I/II and 10 µg/mL DNase I (Sigma, #11284932001), supplemented with 5% fetal bovine serum (FBS, Gibco) at 37°C. The resulting cell suspensions were filtered through a 40‐µm nylon strainer, with red blood cells lysed. Finally, cell viability was assessed using trypan blue solution.

### 4.8 Library Preparation and Sequencing

Beads containing unique molecular identifiers (UMI) and cell barcodes were loaded to near saturation, ensuring that each cell was paired with a bead in a Gel Beads‐in‐emulsion. Afterward, polyadenylated RNA molecules were hybridized to the beads after exposure to cell lysis buffer. The beads were then retrieved into a single tube for reverse transcription. During cDNA synthesis, each cDNA molecule was tagged at the 5' end with both a UMI and a cell label indicating its cell of origin. Sequencing libraries were prepared using randomly interrupted whole‐transcriptome amplification products to enrich the 3' end of the transcripts linked to the cell barcode and UMI. All remaining procedures, including library construction, were performed according to the standard manufacturer's protocol. The libraries were sequenced on the DNBSEQ platform using 2 × 150 chemistry. Sequencing and subsequent bioinformatic analysis were conducted on the platform of Shanghai Majorbio Bio‐pharm Biotechnology Co., Ltd. (Shanghai, China).

### 4.9 Single‐Cell RNA Sequencing Data Processing

Reads were processed with the Cell Ranger pipeline (v7.1.0) using default recommended parameters. The resulting FASTQ files were aligned to the mouse reference genome GRCm38 via the STAR algorithm [60]. Gene‐barcode matrices were then generated for each sample by counting UMIs and filtering out barcodes not associated with cells, yielding a final matrix containing barcoded cells and their corresponding gene expression counts. This output was imported into the Seurat R toolkit (v4.1.1) for quality control and downstream analysis of the single‑cell RNA‑seq data [61].

Cells of low quality were first filtered out based on three standard metrics: (1) number of detected transcripts, (2) number of detected genes, and (3) percentage of reads mapped to mitochondrial genes. Data were normalized using the NormalizeData function in Seurat, after which a subset of variable genes was identified, taking into account the strong dependence of variability on average expression. Finally, cell clusters were visualized on a two‑dimensional map generated by Uniform Manifold Approximation and Projection (UMAP).

### 4.10 Identification of Cell Types and Subtypes

Cell clusters were identified using graph‐based clustering (Louvain algorithm) and annotated with SingleR [62] and known marker genes (Table S2). Chondrocyte subpopulations were further classified according to their expression of both established and novel markers.

### 4.11 Micro‐Computed Tomography (µCT)

TMJ samples were scanned using a Bruker SkyScan1276 µCT system at 9.66 µm resolution (55 kV, 200 µA). Three‐dimensional reconstructions were created using DataViewer, CTan, and CTvox software. The bone morphometric parameters analyzed included bone volume/tissue volume (BV/TV), trabecular thickness (Tb. Th), trabecular number (Tb. N), and trabecular spacing (Tb. Sp).

### 4.12 Histological Analyses

The condylar tissues were collected from mice and fixed in 4% paraformaldehyde for 24 h. Following decalcification and dehydration, the tissues were embedded in paraffin and sectioned into 5 µm sections. H&E staining and Safranine O‐Fast green staining were then performed on the condylar tissue sections according to the manufacturer's protocol. The thickness of condylar cartilage was evaluated by H&E staining, and modified Mankin score (0‐10) [63] was used to quantify the cartilage degeneration by Safranine O‐Fast green staining.

### 4.13 Immunohistochemistry Staining

Immunohistochemical staining was carried out as previously described [64]. After deparaffinization, hydration, and antigen retrieval via microwave, the endogenous peroxidase within tissues was inactivated using H_2_O_2_. Non‐specific interactions were then blocked with goat serum. The sections were incubated with primary antibodies overnight at 4°C, followed by incubation with the secondary antibody and horseradish peroxidase, which were then colored with DAB. Finally, the nuclei were stained with hematoxylin. Positive staining within the condylar cartilage was measured using Image J 1.8.0 software.

### 4.14 Immunofluorescent Staining

Immunofluorescent staining was performed on the condylar tissues of mice. Sections of mouse TMJ tissues underwent heat‐induced antigen retrieval in citrate buffer. Nonspecific interactions were blocked with serum, and the sections were incubated with primary antibodies overnight at 4°C. They were then incubated with DyLight 488/594‐conjugated Goat Anti‐Rabbit/Mouse IgG at 37°C for one hour. The cell nuclei were subsequently stained with 4',6‐diamidino‐2‐phenylindole dihydrochloride (DAPI). Positive immunostaining was quantified by calculating the percentage of positive cells or the average optical density (AOD) of positive staining using ImageJ 1.8.0 software.

### 4.15 Enzyme‐Linked Immunosorbent Assay (ELISA)

The concentration of TGFBI in the TMJ synovial fluid of patients was analyzed using ELISA. Briefly, collected synovial fluid samples were centrifuged to remove sediment and analyzed with a human TGFBI ELISA Kit (HM13288, Bioswamp Life Science Lab) following the manufacturer's protocol. Specially, the total protein concentration in the synovial fluid was determined by bicinchoninic acid analysis, and the final concentration of TGFBI was normalized to the total protein concentration of the TMJ synovial fluid.

### 4.16 Antibodies

The information of antibodies used in this study was provided in the Table S3.

### 4.17 cPCs Isolation and Culture

cPCs were collected from condylar cartilage of rats/mice following previous reported protocols [14, 15]. The condylar tissues were isolated from sacrificed 10–12 weeks‐old rats or mice, as specifically stated. The superficial condylar perichondrium was then carefully dissected using ophthalmic forceps. After cleaning and shredding, the perichondrium samples underwent successive digestion with 0.25% trypsin, followed by collagenase I and dispase II at 37°C. The resulting single cells were subsequently subjected to centrifugation for separation and cultured in DMEM medium supplemented with 20% FBS, 1× glutamax, 55 mM 2‐mercaptoethanol, and 1% penicillin‐streptomycin.

Cultured cPCs were detached using trypsin and then plated at passages 1–2 for in vitro studies. Unless otherwise specified, the cPCs were treated with the following reagents and concentration: recombinant human TGFBI (rhTGFBI, 200 ng/ml), IL‐1β (20 ng/ml), H_2_O_2_ (100 µM), OSMI‐1 (OSMI, 10 µM), Thiamet G (TMG, 10 µM).

### 4.18 Chondrogenic Differentiation of cPCs

To induce the chondrogenic differentiation of cPCs, the following culture medium were used [14]: DMEM medium with 10% FBS, 0.1 µM dexamethasone, 1% insulin transferrin selenium (ITS), 100 µM ascorbic acid, 10 ng/ml TGF‐β1, and 1 mM sodium pyruvate. For monolayer culture, cPCs were inoculated onto a well plate and induced for 5 to 7 days. To perform the centrifuge tube spheroid formation culture, 10^6^ cPCs were inoculated into a 15 mL centrifuge tube and induced for 7 to 14 days, with the culture medium exchanged every other day. For spheroid formation in Matrigel, digested cPCs were inoculated into undiluted Corning Matrigel, and the induction culture medium was added after the Matrigel had cured, followed by a 14‐day incubation period.

### 4.19 cPCs Spheroids Processing and Staining

cPCs spheroids were obtained from both centrifuge tube spheroid formation culture and Matrigel spheroid formation culture. The collected cPCs spheroids were embedded in optimal cutting temperature (OCT) compound and sectioned into 6 um slices using a freezing microtome (Leica). Following hydration, the spheroid slices underwent Safranine O staining according to the manufacturer's instructions. For immunofluorescent staining, the hydrated slices were first incubated with goat serum to block nonspecific interactions, then incubated with primary antibodies overnight at 4°C. The following day, the slices were incubated with DyLight 488‐conjugated or DyLight 594‐conjugated secondary antibodies for 1 h. After mounting with an anti‐fluorescence quenching medium containing DAPI, the positive staining was visualized using confocal microscopy (Olympus, FV1200).

### 4.20 Bulk RNA Sequencing

Bulk RNA sequencing was conducted to analyze the transcriptional changes in cPCs after the induction of O‐GlcNAcylation. Total RNA was extracted from rat cPCs that were treated with or without 10 µM Thiamet G (MCE, HY‐12588) using TRIzol reagent, three independent biological replicates were performed for each group. RNA libraries were then prepared and sequenced on a NovaSeq X Plus platform (paired‐end 150 bp) by Shanghai Majorbio Bio‐pharm Biotechnology. The raw reads underwent quality control using fastp and were subsequently aligned to the reference genome. Finally, transcript assembly was performed using StringTie [65].

Gene expression levels were quantified in transcripts per million (TPM). Differentially expressed genes (DEGs) were identified using DESeq2, with a threshold of |log_2_ fold change| ≥ 1 and p < 0.05 considered statistically significant. To further analyze these genes, Gene Ontology (GO) and Kyoto Encyclopedia of Genes and Genomes (KEGG) enrichment analyses were performed on the cloud platform of Majorbio Bio‐pharm Biotechnology.

### 4.21 Lentivirus Packaging and Transfection

Lentivirus transfection of small hairpin RNA (shRNA) was used for *Adamts16* knockdown in mouse cPCs. Lentivirus shAdamts16 (Lv‐sh*Adamts16*) and a nontargeting lentiviral vector (Lv‐vector) were designed, synthesized and packaged by shanghai Genechem Co., Ltd. Lv‐sh*Adamts16* and Lv‐vector was diluted to an MOI of 20 [66], mixed with a special transfection agent from Genechem Co., Ltd for 15 min, and then added to cultured mouse cPCs. The transfection medium was replaced with culture medium 24 h after transfection. The knockdown efficiency was assessed using qRT‐PCR analysis 36 h after transfection and western blotting 48 h post‐transfection.

### 4.22 SA‐β‐Gal Staining

Following the appropriate treatment, cultured cPCs were washed twice with PBS and fixed in a 4% PFA fixation solution for 10 min. The fixed cells were then washed and incubated in SA‐β‐Gal staining solution overnight at 37°C. After incubation, the cells were washed twice with PBS and mounted with a water‐soluble mounting medium. Stained cells were imaged under a bright‐field microscope (Leica), and both SA‐β‐Gal positive cells and total cells were counted in three random fields for each biological replicate.

### 4.23 RNA Isolation and qRT‐PCR

Cells were collected and treated with TRIzol reagent for total RNA extraction, which was subsequently reverse transcribed into cDNA using a reverse transcription kit (TaKaRa, Japan). The reverse transcribed cDNA was amplified using quantitative polymerase chain reaction (PCR) with ChamQ SYBR qPCR Master Mix (Vazyme, Nanjing). The cycle threshold method (2^−∆∆Ct^) was then employed to quantify the mRNA levels of each analyzed gene, normalizing the measurements to control groups set to 1 in each biologically independent trial. The sequences of the primers used in this study were listed in Table S4.

### 4.24 Western Blotting

Protein extraction was performed with RIPA buffer containing cOmplete protease inhibitor cocktail. Following extraction, proteins were resolved by polyacrylamide gel electrophoresis and transferred onto PVDF membranes via electroblotting. The membranes were blocked with skim milk and subsequently probed with primary antibodies at 4°C overnight. After washing, incubation was carried out with HRP‐conjugated goat anti‐rabbit or goat anti‐mouse secondary antibodies. Protein bands were detected using an Odyssey Fc Imaging System after application of ECL reagents. All experiments were performed in three independent replicates.

### 4.25 Succinylated Wheat Germ Agglutinin (sWGA) Pulldown

Cells treated with Thiamet G were lysed using IP lysis buffer and incubated at 4 °C for 30 min. After washing with washing buffer, succinylated wheat germ agglutinin (sWGA)‐conjugated agarose beads (Vector Laboratories, #AL‐1023S‐2) were incubated with the pre‑cleared lysates overnight at 4 °C. The following day, precipitated complexes were eluted three times with washing buffer, mixed with immunoblot loading buffer, and denatured at 95 °C for 10 min. Finally, the supernatants of the precipitated complexes were subjected to immunoblotting with the indicated antibodies.

### 4.26 CHX Chase Analysis

ATDC5 chondrogenic cells were treated with Thiamet G for 48 h, followed by exposure to 100 µg//ml cycloheximide (CHX, HY‐12320, MCE) for specified durations (0, 1, 2, 4, and 8 h). After protein harvesting, endogenous ADAMTS16 protein levels were measured by immunoblotting to analyze the effect of O‐GlcNAcylation on ADAMTS16 protein stability.

### 4.27 Prediction of O‐GlcNAcylation Sites

Potential O‐GlcNAcylation sites on ADAMTS16 were predicted using the NetOGlyc‐4.0 website (https://services.healthtech.dtu.dk/services/NetOGlyc‐4.0/). Sites with an output prediction value>0.9 in mouse *Adamts16* CDS sequences were selected for further analysis. Additionally, a cross‐species alignment was conducted to assess the conservation of O‐GlcNAcylation sites.

### 4.28 Plasmid Construct and Transient Transfection

The plasmids utilized in this study, including Flag‐vector, Flag‐m*Adamts16*‐WT, Flag‐m*Adamts16*‐S989A, and Flag‐m*Adamts16*‐S1170A, were constructed by Miao Ling Plasmid Platform (Wuhan, China). A Quik Change Site‐Directed Mutagenesis Kit (Agilent, #200518) was employed for site‐directed mutagenesis of the mouse Adamts16 gene.

Transient transfection of plasmids was performed using Lipofectamine 2000 Transfection Reagent (Invitrogen, #11668019). Briefly, plasmids and transfection reagent were diluted in Opti‐MEM respectively. After 5 min, the two solutions were mixed and allowed to stand for 15 min to form a transfection complex. This complex was then added to HEK‐293T cells, and replaced with fresh complete medium after 6 h.

### 4.29 Molecular Docking

The AlphaFold 3 model was utilized to forecast protein‐protein interactions. Amino acid sequences were obtained from UniProt (https://www.uniprot.org/) and subsequently uploaded to the AlphaFold 3 platform for structural prediction.

To ensure docking accuracy, protein structures were prepared with AutoDockTools‐1.5.7, which included the manual removal of water molecules and addition of polar hydrogen atoms. Protein‐protein docking was then conducted using the GRAMM web server. The resulting complexes were further refined in AutoDockTools‐1.5.7 by repeating the removal of water and addition of polar hydrogens. Finally, protein‐protein interactions were analyzed and visualized with PyMOL.

### 4.30 Co‐Immunoprecipitation (Co‐IP)

Co‐immunoprecipitation was carried out using the Pierce Classic Magnetic IP/Co‑IP Kit (Thermo Scientific, #88804) according to the manufacturer's instructions. Cell lysates prepared in IP lysis buffer were incubated overnight at 4 °C with an anti‑Flag antibody. The following day, the antigen‑antibody complexes were captured by incubation with Protein A/G magnetic beads for 1 h at room temperature. After washing twice with IP Lysis/Wash Buffer and once with purified water, the bound complexes were eluted and subsequently analyzed by western blotting.

### 4.31 Cellular Immunofluorescence

Cells were fixed with 4% paraformaldehyde and permeabilized with 0.5% Triton X‐100 when necessary. Following blocking with bovine serum albumin (BSA), samples were incubated with primary antibodies at 4 °C overnight. The next day, cells were stained with fluorophore‑conjugated secondary antibodies and mounted using an anti‐fade mounting medium containing DAPI. For immunofluorescence co‐staining, cells were incubated with another primary antibodies at 4 °C overnight after fluorophore‑conjugated secondary antibodies staining. And stained with another secondary antibodies on the other day, and mounted using mounting medium containing DAPI. Immunofluorescence images were acquired under a fluorescence microscope and analyzed with ImageJ software (version 1.8.0).

### 4.32 Statistics

All data analysis in this study was conducted using GraphPad Prism 8.0 software. The Shapiro‐Wilk test was employed to assess the normality of the data, while F tests were used to evaluate variance equality. As specified in the figure legends, statistical analysis was performed using Student's t test, one‐way analysis of variance (ANOVA) followed by Dunnett's multiple comparisons test, or two‐way ANOVA followed by Sidak's multiple comparisons test. In the statistical analysis, a *p* value of less than 0.05 was considered statistically significant.

## Author Contributions

Conceptualization: **X. Liu**, **X. Luo**, **J. Ke**, **X. Long**; Methodology: **X. Liu**, **X. Luo**; Investigation: **X. Liu**, **X. Luo**; Software: **X. Liu**, **X. Luo**, **J. Zhao**, **Z. Jiang**; Visualization: **X. Liu**, **J. Zhai**, **T. Zhu**, **X. Jia**, **X. Ma**; Formal analysis: **X. Liu**, **H. Jiang**, **H. Li**, **Y. Feng**; Data curation: **X. Liu**, **X. Luo**, **J. Ke**, **X. Long**; Funding acquisition: **X. Liu**, **X. Luo**, **J. Ke**, **X. Long**; Supervision: **X. Liu**, **J. Ke**, **X. Long**; Writing – original draft: **X. Liu**, **X. Luo**; Writing – review & editing: **X. Liu**, **J. Ke**, **X. Long**.

## Funding

National Natural Science Foundation of China grant 82301114 (X. Liu) and 82403457 (X. Luo) and 82170983 (X. Long); National Key Research and Development Project of China grant 2023YFC2509200 (J. Ke); Natural Science Foundation of Hubei Province grant 2023AFB065 (X. Liu); Fundamental Research Funds for the Central Universities grant 2042023kf0145 (X. Liu).

## Conflicts of Interest

The authors declare no conflicts of interest.

## Supporting information




**Supporting File**: advs77594‐sup‐0001‐SuppMat.docx.

## Data Availability

The data that support the findings of this study are available from the main text, the supplementary materials, or from the corresponding author upon reasonable request.
