# Supplementary material for: TGFBI Maintains Fibrocartilage Homeostasis by Restraining O‐GlcNAcylation to Inactivate and Destabilize ADAMTS16 in Temporomandibular Joint Osteoarthritis
Source: Adv Sci (Weinh). 2026 Sep 16:e77594. Online ahead of print. doi: 10.1002/advs.77594 (PMC13583117; doi:10.1002/advs.77594)
Supplement: Supplementary file 1 [file ADVS-9999-e77594-s001.docx]

Supplementary Materials

**TGFBI maintains fibrocartilage homeostasis by restraining O-GlcNAcylation to inactivate and destabilize ADAMTS16 in temporomandibular joint osteoarthritis**

Xin Liu *et al*

**Fig. S1 to S9**

**Table. S1 to S5**

Fig. S1. Expression of integrin receptors in human TMJOA cartilage and mouse condylar cartilage

(**A**) Immumohistochemical staining of integrin αV/β3 and αV/β5 in the human TMJOA condylar cartilage. (**B-D**) Violin plots demonstrating expression levels of integrin *Itgav*, *Itgb3*, and *Itgb5* in different cell types from mouse condylar tissues.

Fig. S2. Validation of *Tgfbi* knockout and cartilage biology of **WT and *Tgfbi*^-/-^** mice

(**A**) Sanger sequencing confirmation of the *Tgfbi* knockout region in *Tgfbi*^-/-^ mice. (**B**) gRNA targeting strategy and genotyping primer design (F1, F2, R1) for *Tgfbi* knockout. (**C**) Representative genotyping results for *Tgfbi*^+/+^, *Tgfbi*^+/-^, and *Tgfbi*^-/-^ mice. (**D**) Representative H&E staining images of condylar cartilage from age-related WT and *Tgfbi*^-/-^ mice. (**E**) Quantitative analysis of cartilage thickness and number of chondrocytes from (D), unpaired t-test, n=6. (**F**) Violin plots demonstrating expression levels of integrin *Itgb5*, *Itgb3*, and *Itgav* in the cell clusters from condylar cartilage of WT and *Tgfbi*^-/-^ mice. Data are presented as mean ± SD; **p < 0.01, ns, not significant.

Fig. S3. Single-cell transcriptomic characterization of mouse condylar chondrocytes

(**A**) UMAP visualization of all cell clusters from condylar cartilage and surrounding tissues, red box highlights chondrocytes. (**B**) UMAP feature plots of chondrocyte markers *Col2a1*, *Sox9*, *Acan*, and *Comp*. (**C**) UMAP plots comparing eight chondrocyte clusters between WT and KO mice. (**D**) Proportional distribution of 8 chondrocyte clusters in WT versus KO groups. (**E**) UMAP expression plots of proliferative markers *Mki67*, *Top2a*, and *Ube2c*. (**F**) Bubble chart of marker genes for proliferating chondrocytes in eight chondrocyte clusters. (**G-H**) Monocle2 pseudotime analysis showing differentiation trajectories of eight chondrocyte clusters. (**I**) Violin plots exhibiting expression levels of integrin *Itgav*, *Itgb3*, and *Itgb5* across four chondrocyte subtypes.

Fig. S4. TGFBI regulation of senescence and metabolic markers in rat cPCs

(**A**) Flow chart of condylar perichondrium collection and primary culture process for cPCs. (**B**) Western blot analyzing TGFBI expression in rat cPCs after rhTGFBI treatment. (**C**) Schematic illustrating the role of CDK inhibitors (Cdkn1a, Cdkn2a, Cdkn1b) in cell cycle arrest, and qRT-PCR analysis of *Cdkn1a*, *Cdkn2a*, and *Cdkn1b* mRNA levels in rat cPCs treated with rhTGFBI, unpaired t-test, n=3. (**D-E**) Immunofluorescence staining and quantitative analysis for the expression of integrin αV/β3 and αV/β5 in rat cPCs, unpaired t-test, n=6. (**F-G**) qRT-PCR analyzing the mRNA levels of catabolic and anabolic markers in rat cPCs, one-way ANOVA, n=3. Data are presented as mean ± SD; *p < 0.05, **p < 0.01, ***p < 0.001; ns, not significant. cPCs, condylar perichondrial cells.

Fig. S5. TGFBI regulates condylar chondrocyte catabolism and senescence, and subchondral bone microstructure in TMJOA mice

(**A**) Representative immunohistochemical staining of catabolic mediators (ADAMTS5, MMP13) and senescence markers (p16, p21) in mouse condylar cartilage, (**B**) Quantitative analysis of immunohistochemical staining in (A), one-way ANOVA, n=6. (**C-D**) Representative immunofluorescence staining and quantification of TGFBI in the condylar cartilage of TMJOA mice, with or without AAV-*Tgfbi* injected, unpaired t-test, n=6. (**E-F**) Immunohistochemical staining (E) and quantification (F) of catabolic mediators (ADAMTS5, MMP13) and senescent factors (IL-1β, IL-6) in mouse condylar cartilage, one-way ANOVA, n=6. (**G-H**) Representative micro-CT images (G) and quantitative analysis (H) of subchondral bone microstructure, one-way ANOVA, n=6. Data are presented as mean ± SD; *p < 0.05, **p < 0.01, ***p < 0.001; ns, not significant. AOD, average optical density.

Fig. S6. O-GlcNAcylation and matabolic-related biomarkers in rat cPCs, human and mouse condylar cartilage

(**A**) Violin plot showing expression levels of *Col2a1* across four chondrocyte subtypes. (**B**) Violin plots showing expression levels of *Oga* and *Col2a1* in chondrocyte clusters from WT and KO mice. (**C**) H&E staining of human TMJOA condylar cartilage, and representative RL2 immunohistochemical staining images of undamaged and damaged area in human condylar cartilage. (**D-E**) qRT-PCR analysis of catabolic markers (*Adamts5*, *Mmp13*, *Col10a1*) and anabolic markers (*Acan*, *Col2a1*, *Sox9*) in rat cPCs under indicated treatments, unpaired t-test, n=3. (**F-G**) Immunohistochemical staining (F) and quantification (G) of O-GlcNAc and OGT in condylar cartilage from TMJOA mice, one-way ANOVA, n=6. (**H**) qRT-PCR analysis of *Oga* and *Ogt* mRNA levels in rhTGFBI-treated rat cPCs, unpaired t-test, n=3. Data are presented as mean ± SD; *p < 0.05, **p < 0.01, ***p < 0.001. cPCs, condylar perichondrial cells.

Fig. S7. Quality control, differential expression analysis, and knockdown validation of bulk RNA-seq

(**A**) Gene expression distribution in control and TMG-treated rat cPCs. (**B**) PCA plot of six samples (n=3 per group). (**C**) Correlation heatmap between six samples of rat cPCs. (**D**) Venn diagram of co-expressed and uniquely expressed genes. (**E**) Statistics of up- and down-regulated DEGs (p<0.05, |log2FC|≥1). (F) MA plot of DEGs between two groups. (**G**) Circular heatmap of DEGs between control and TMG-treated cPCs. (**H**) Western blot and qRT-PCR validation of *Adamts16* knockdown efficiency in mouse cPCs after Lv-sh*Adamts16* transfection, unpaired t-test, n=3 in western blot, and n=4 in qRT-PCR. (**I-J**) qRT-PCR analysis of senescent factors (*Cdkn1a*, *Cdkn2a*, *Cdkn1b*) and catabolic markers (*Adamts5*, *Mmp13*) in mouse cPCs under indicated treatments, one-way ANOVA, n=3. Data are presented as mean ± SD; *p < 0.05, **p < 0.01, ***p < 0.001. cPCs, condylar perichondrial cells; PCA, principal component analysis; DEGs, differentially expressed genes; SASP, senescence-associated secretory phenotype.

Fig. S8. Fibrosis, catabolic and senescence factors in *Tgfbi*-deficient TMJOA mice treated with OSMI-1 or Lv-sh*Adamts16*

(**A-B**) Immunofluorescence staining (A) and quantification (B) of Collagen I-positive cells in condylar perichondrium of MIA-induced TMJOA mice, one-way ANOVA, n=6. (**C-D**) Immunohistochemical staining (C) and quantification (D) of catabolic mediators (ADAMTS5, MMP13) and senescence markers (p21, p16) in mouse condylar cartilage, one-way ANOVA, n=6. (**E-F**) Immunohistochemical staining (E) and quantitative analysis (F) of O-GlcNAc and OGT in mouse condylar cartilage, one-way ANOVA, n=6. Data are presented as mean ± SD; *p<0.05, **p < 0.01, ***p < 0.001.

Fig. S9. ADAMTS16 expression in *Tgfbi*-deficient TMJOA mice and O-GlcNAcylation site mapping

(**A-B**) Immunohistochemical staining (A) and quantification (B) of ADAMTS16 in condylar cartilage from WT and *Tgfbi*-deficient TMJOA mice, one-way ANOVA, n=6. (**C**) NetOGlyc-4.0 prediction of O-GlcNAcylation sites on mouse *Adamts16* (score > 0.9). (**D**) Cross-species sequence alignment for conserved O-GlcNAcylation sites. (**E-F**) Sequencing confirmation of site-directed mutants: *Adamts16*-S989A (E) and *Adamts16*-S1170A (F). Data are presented as mean ± SD; *p < 0.05, **p < 0.01.

| **Stage** | **Condylar shape and height** | **Schematic diagram** |
| --- | --- | --- |
| 0 | Normal condylar shape and height |  |
| 1 | Normal condylar height with local mild condylar resorption |  |
| 2 | Reduced condylar height with moderate condylar resorption |  |
| 3 | Severe condylar resorption, presenting the basic shape of condyle |  |
| 4 | Severe condylar resorption, losing the basic shape of condyle |  |

**Table S1. Staging system for human condylar shape and height based on CBCT**

| **Clustering** | **Biomarkers** |
| --- | --- |
| B_cell | *Cd19; Cd79a; Ms4a1* |
| Chondrocyte | *Col2a1; Sox9; Acan; Comp* |
| Endothelial | *Vwf; Cldn5; Pecam1* |
| Erythroblast | *Hba-a1; Hba-a2; Hbb-bs; Hbb-bt; Gypa* |
| Mast_cell | *Tpsb2; Cma1; Hpgds; Tpsab1; Cpa3* |
| Macrophage | *Cd68; Cd163; Adgre1; Aif1; c1qa* |
| NK_cell | *Nkg7; Klrd1* |
| T_cell | *Cd3d; Cd3g; Cd3e* |
| Neutrophil | *Csf3r; S100a8; G0s2; Cxcr2* |

Table S2. Biomarkers using for single-cell clustering analysis

| **Antibodies** | **Species** | **Source** | **Catalog No.** | **Applications** |
| --- | --- | --- | --- | --- |
| TGFBI | Rabbit | Abcam | ab170874 | WB, IHC |
| Integrin αV/β3 | Mouse | Invitrogen | 14-0519-82 | IHC, IF |
| Integrin αV/β5 | Mouse | Abcam | ab177004 | IHC, IF |
| ACAN | Rabbit | Proteintech | 13880-1-AP | IF |
| SOX9 | Rabbit | Abcam | ab185230 | IF, WB |
| Ki67 | Rabbit | Abcam | ab15580 | IF |
| Collagen I | Rabbit | Proteintech | 14695-1-AP | IF |
| ADAMTS5 | Rabbit | Abcam | ab41037 | IHC, WB |
| MMP13 | Rabbit | Proteintech | 18165-1-AP | IHC, WB |
| IL-1β | Rabbit | Abcam | ab9722 | IHC |
| IL-6 | Rabbit | Servicebio | GB11117 | IHC |
| S100A8 | Rabbit | Servicebio | GB11421 | IHC |
| S100A9 | Rabbit | Servicebio | GB111149 | IHC |
| p21 | Rabbit | Servicebio | GB155313 | IHC |
| p16 | Rabbit | Servicebio | GB151143 | IHC |
| O-GlcNAc/RL2 | Mouse | Abcam | ab2739 | WB, IHC |
| OGT | Rabbit | Abcam | ab177941 | WB, IHC |
| OGT | Mouse | Proteintech | 66823-1-Ig | IF |
| OGA | Rabbit | Proteintech | 14711-1-AP | WB |
| ADAMTS16 | Rabbit | Cusabio | PA244586 | WB, IHC |
| Flag-tag | Mouse | Abmart | M20008 | WB, IP |
| NLRP3 | Rabbit | Abcam | ab263899 | WB |
| MMP3 | Rabbit | Immunoway | YT4465 | WB |
| iNOS | Mouse | Invitrogen | MA5-17139 | WB |
| COX2 | Rabbit | Cell Signaling | #12282 | WB |

Table S3. Antibodies information

WB, Western Blot; IHC, Immunohistochemistry; IF, Immunofluorescence; IP, Immunoprecipitation.

| **Gene** | **Forward** | **Reverse** |
| --- | --- | --- |
| m_*β-actin* | CACTGTCGAGTCGCGTCC | CGATGGAGGGGAATACAGCC |
| m_*Adamts16* | AGTCCTATAGGCCGGGACTG | ATTACTGCACTCGCTCCCAC |
| m_*Cdkn1a* | CAGAATAAAAGGTGCCACAGGC | CGTCTCCGTGACGAAGTCAA |
| m_*Cdkn2a* | TGAATCTCCGCGAGGAAAGC | TGCCCATCATCATCACCTGAA |
| m_*Cdkn1b* | CAGACGTAAACAGCTCCGAATTA | GGCAGATGGTTTAAGAGTGCC |
| m_*Adamts5* | GGAGCGAGGCCATTTACAAC | CGTAGACAAGGTAGCCCACTTT |
| m_*Mmp13* | CTTCTTCTTGTTGAGCTGGACTC | CTGTGGAGGTCACTGTAGACT |
| r_*β-actin* | GCAGGAGTACGATGAGTCCG | ACGCAGCTCAGTAACAGTCC |
| r_*Cdkn1a* | AGACAACAAGGGCAAGATGGT | GGAACAGGTCGGACATCACC |
| r_*Cdkn2a* | GACACTCTGGTAGTACTGCACC | GCTTGAGCAGAAGTTATGCCTG |
| r_*Cdkn1b* | TCGCAAAACAAAAGGGCCAA | AGGAATTATTCTTAATTCGGAGCTG |
| r_*Adamts5* | CCCAAATACGCAGGTGTCCT | ACACACGGAGTTGCTGTAGG |
| r_*Mmp13* | TCCATCCCGAGACCTCATGT | CTCAAAGTGAACCGCAGCAC |
| r_*Col10a1* | TCTAAAGTGCACTCCGGGAC | AAACGAGATTCATGATGCGCT |
| r_*Acan* | ATGCCTTCCGAGCTACAGAG | GCAGGGAGTGTCCATCAGAC |
| r_*Col2a1* | TCCTACAATGTCAGGGCCAG | TCATCGCAGAGGACATTCCC |
| r_*Sox9* | CTCCTACTACAGCCACGCAG | AGCTGTGTGTAGACGGGTTG |
| r_*Oga* | GGCAGTAGCTGCTTAGGGAC | GCCAAGAGACTAAGCGGAGG |
| r_*Ogt* | TATTCTGCCACCCCCTAAGCC | TGCCAACTCAGCTAACCCTTG |

Table S4. Primer sequences

| **Reagent** | **Source** | **Catalogue No.** |
| --- | --- | --- |
| MIA | Sigma-Aldrich | #57858 |
| Safranine O | Sigma-Aldrich | S8884 |
| Fast green | Sigma-Aldrich | F7252 |
| UltraSensitive SP | MXB Biotechnologies | KIT-9706 |
| OSMI-1 | MedChemExpress | HY-119738 |
| Thiamet G | MedChemExpress | HY-12588 |
| DNase I | Sigma-Aldrich | #11284932001 |
| Collagenase type I | Biosharp | BS163 |
| Collagenase type II | Biosharp | BS164 |
| Dispase II | Solarbio | D6430 |
| Glutamax | Gibco | #35050061 |
| 2-Mercaptoethanol | Macklin | M6230 |
| Penicillin-streptomycin | Gibco | #15140122 |
| rhTGFBI | Abnova | P3529 |
| IL-1β | PeproTech | #200-01B |
| H_2_O_2_ | Sigma-Aldrich | #323381 |
| Dexamethasone | MedChemExpress | HY-14648 |
| Insulin transferrin selenium | Sigma-Aldrich | I3146 |
| Ascorbic acid | Sigma-Aldrich | A4544 |
| TGF-β1 | PeproTech | #100-21C |
| Sodium Pyruvate | Gibco | #11360070 |
| Matrigel | Corning | #356234 |
| sWGA-conjugated agarose beads | Vector Laboratories | AL-1023S-2 |
| Cycloheximide | MedChemExpress | HY-12320 |
| Lipofectamine™ 2000 | Invitrogen | #11668019 |
| Pierce™ Classic Magnetic Co-IP Kit | Thermo Scientific | #88804 |

Table S5. Reagents Catalogue
